# Large-scale multi-omics unveils host–microbiome interactions driving root development and nitrogen acquisition

**DOI:** 10.1038/s41477-025-02210-7

**Published:** 2026-02-03

**Authors:** Nannan Li, Guoliang Li, Xiaofang Huang, Lige Ma, Danning Wang, Yu Luo, Xulv Cao, Yantao Zhu, Jianxin Mu, Ran An, Jianhua Zhao, Yongfeng Wang, Cuiling Yang, Hao Chen, Ying Xu, Lixi Jiang, Meng Luo, Xiaodan Li, Yachen Dong, Xinping Chen, Frank Hochholdinger, Yong Jiang, Jochen C. Reif, Daojie Wang, Yanfeng Zhang, Yang Bai, Peng Yu

**Affiliations:** 1 College of Resources and Environment, and Academy of Agricultural Sciences, Southwest University, Chongqing, China; 2 Leibniz Institute of Plant Genetics and Crop Plant Research (IPK), Seeland, Germany; 3 Emmy Noether Group Root Functional Biology, Institute of Crop Science and Resource Conservation (INRES), University of Bonn, Bonn, Germany; 4 Plant Genetics, TUM School of Life Sciences, Technical University of Munich (TUM), Freising, Germany; 5 Peking-Tsinghua Center for Life Sciences, State Key Laboratory of Gene Function and Modulation Research, Peking-Tsinghua-NIBS Graduate Program, College of Life Sciences, Peking University, Beijing, People’s Republic of China; 6 Plant Breeding, TUM School of Life Sciences, Technical University of Munich, Freising, Germany; 7 Hybrid Rapeseed Research Center of Shaanxi Province, Yangling, People’s Republic of China; 8 College of Agriculture, State Key Laboratory of Crop Stress Adaptation and Improvement, Henan University, Kaifeng, People’s Republic of China; 9 Institute of Crop Science, Zhejiang University, Hangzhou, People’s Republic of China; 10 Shanghai Majorbio Research Institute, Shanghai, People’s Republic of China; 11 Interdisciplinary Research Center for Agriculture Green Development in Yangtze River Basin, Southwest University, Chongqing, People’s Republic of China; 12 Crop Functional Genomics, Institute of Crop Science and Resource Conservation (INRES), University of Bonn, Bonn, Germany

**Keywords:** Plant development, Genetic association study, Microbiology, Plant genetics

## Abstract

The rhizosphere microbiome plays a crucial role in determining plant performance and fitness. Nevertheless, regulatory mechanisms linking host genetic variation, root gene regulation and microbiome assembly—and their collective influence on plant nutritional traits—remain poorly understood. Here we generated and integrated 1,341 paired datasets, including root transcriptomes, rhizosphere bacterial 16S rRNA profiles and root ionomes, across 175 resequenced *Brassica napus* ecotypes grown at two contrasting field sites. We identified 203 highly heritable bacterial amplicon sequence variants (ASVs), many of which were significantly associated with root nitrogen (N) levels. Host transcriptome-wide gene expression and these microbial features together explained up to 45% of natural variation in N uptake while genome-wide association analyses revealed host loci regulating ASV abundance, many of which were under the control of eQTL hotspots linked to carbon and N metabolism. Isolate-level inoculation, whole-genome sequencing, metabolite profiling and confocal imaging demonstrated that the dominant, genetically regulated bacterial genus *Sphingopyxis* modulates auxin biosynthesis and promotes lateral root development to enhance N acquisition under stress. This study therefore identifies *Sphingopyxis* as a functionally relevant taxon with potential for microbiome-assisted breeding of nutrient-efficient crops.

## Abstract (web summary)

This study uncovers genetic links between plant roots and their microbiome in *Brassica napus*, identifying microbe-associated loci and a beneficial bacterium, *Sphingopyxis*, that promotes nitrogen uptake.

## Main

The rhizosphere, defined as the soil region surrounding plant roots, is a unique and dynamic environment where complex biological interactions occur between the plant root system and the diverse array of soil microbes collectively known as the rhizosphere microbiome^1^. The rhizosphere microbiome influences root traits and functions that directly impact plant health^2^, contributes to nutrient homeostasis in the host^3^, offers protection against both biotic and abiotic stresses^4^, modulates the plant’s developmental program^5^ and is a key driver of broader ecosystem functioning^6^. Microbes residing in the rhizosphere and on plant roots harbour a diverse range of functional traits, including metabolic properties^7–10^ and immune system-related functions^11,12^. Even small host-mediated changes in the microbiome can substantially affect host fitness^13^. Interestingly, while the host genotype does influence the microbiome, its effect is relatively modest compared to the strong impact of soil edaphic factors^14,15^. Genome-wide association studies (GWAS) provide an efficient means to identify genetic variations associated with specific microbial communities^16^. Quantitative analyses of microbiome composition, along with plant genetic diversity, have facilitated the identification of heritable microbial traits, enabling their incorporation into GWAS as potential phenotypes^17–23^. Nevertheless, despite these advancements, a comprehensive understanding of how the rhizosphere microbiome forms and is regulated by host gene expression beyond its influence on plant fitness and agroecosystem functions species remains elusive in crops.

Nitrogen is the most important macro-mineral element for plants in terms of quantity, playing a critical role in crop yield and overall plant health. However, the excessive use of nitrogen fertilizers in agriculture has become a global environmental concern, posing dramatic threats to ecosystems and human health^24,25^. Over the past decades, nitrogen fertilizer application to terrestrial soils has contributed to ~60% of the increase in atmospheric nitrous oxide emissions, a potent greenhouse gas^26,27^. Mineral nutrients, including nitrogen, are absorbed by plants through their elaborate root systems, which interact with the surrounding rhizosphere^28^. Numerous studies have shown that beneficial symbiotic relationships between plant roots and rhizosphere microbes can enhance plant performance by improving nutrient uptake, including nitrogen^29,30^. Growing genetic evidence suggests that the root microbiome plays a pivotal role in enhancing plant nutrient uptake, for example, nitrogen acquisition^31–33^. Furthermore, the microbiome is involved in directly sensing phosphate stress in the soil, as shown in research on plant–microbe interactions^34^. These findings highlight the importance of the rhizosphere microbiome in regulating nutrient dynamics, with implications for both sustainable agriculture and minimizing the environmental impact of fertilizer use.

In this context, understanding the genetic basis and regulatory variation of host–microbiome associations is crucial for deciphering the mechanisms that underlie the formation and function of the crop microbiome, particularly in relation to plant nutrition. The present study takes an integrative approach, combining rhizosphere microbiome analyses with host GWAS and transcriptome-wide association studies (TWAS) across two independent environments. By quantifying the interactions among genotypes, environmental factors and plant nutritional status, the study aims to predict the overall fitness and health of rapeseed (*Brassica napus*) plants. The findings provided insights into the beneficial associations between the soil microbiome and the plant root system, offering a pathway for developing crop varieties with optimized root phenotypes. Such varieties would be designed to recruit and activate microbiomes that confer a range of benefits, including enhanced crop productivity, more efficient nutrient acquisition and improved agroecosystem resilience. By leveraging the genetic basis, gene regulation and microbiome interactions identified in this study, it may be possible to improve crop health and sustainability in agricultural systems, ultimately contributing to more efficient and eco-friendly farming practices.

## Results

### Integrative multi-omics analyses of the rapeseed genome, root transcriptome and rhizosphere bacterial microbiome

**Fig. 1:**
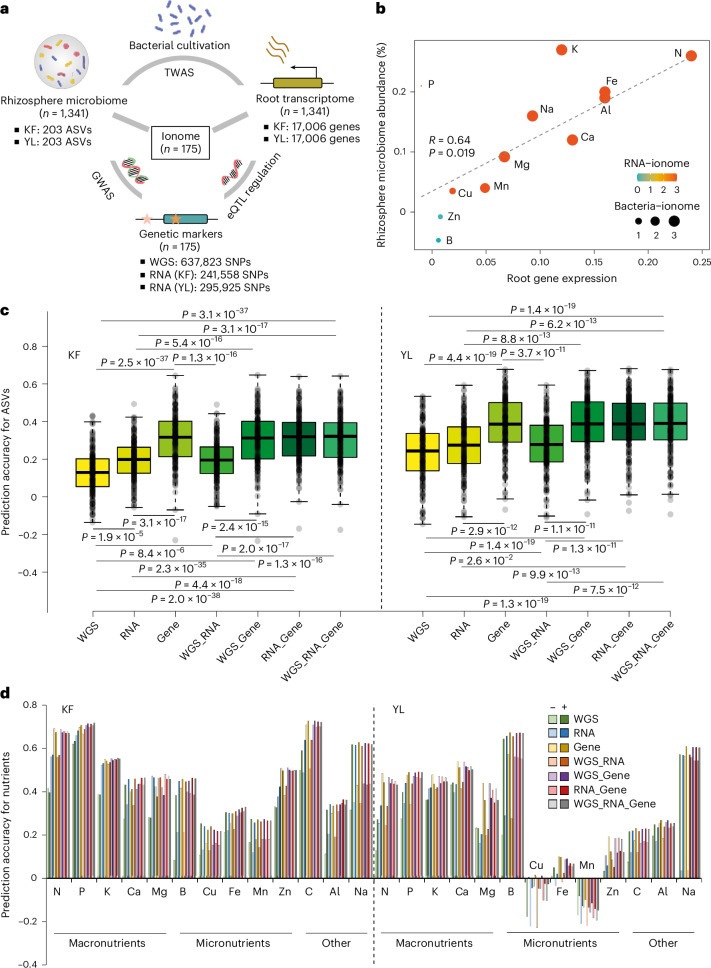
Genomic and transcriptomic analyses of host–bacterial microbiome association and plant nutritional traits in *B. napus.* **a**, A schematic overview of omics datasets used in this study. WGS SNPs data for rapeseed accessions (*n* = 175) were derived from ref. ^44^. RNA-seq SNP data were extracted from RNA sequencing results for both KF and YL locations. Ionome data include the concentration of 12 different mineral nutrients for 175 rapeseed accessions. In total, 1,341 samples were generated for both root RNA-seq and rhizosphere microbiome together with another bulk soil (*n* = 72) microbiome analysis. **b**, Spearman correlation between gene expression, rhizosphere bacterial microbiome diversity and plant ionomic traits. The *x* axis shows the correlation coefficient between root gene expression and ionomic traits, and the *y* axis shows the corresponding correlation between rhizosphere bacterial abundance (relative abundance; *n* = 203 ASVs) and the same ionomic traits. Point colour indicates the significance of RNA–ionome correlations (−log_10_ *P*), and point size indicates the significance of bacteria–ionome correlations (−log_10_ *P*). The dashed line represents the linear regression fit (Spearman’s *R* = 0.64, *P* = 0.019). **c**, Different scenarios for predicting the abundance of bacterial ASVs in the rhizosphere bacterial microbiome. Boxplots show the distribution of prediction accuracy for each ASV, with individual ASVs represented as overlaid dot plots. Accuracy was computed using models trained on different combinations of omics data: WGS SNPs, RNA-seq SNPs, gene expression and their pairwise or three-way integrations. The *y* axis indicates prediction accuracy (*r*). Statistical comparisons between prediction scenarios were performed using two-sided Wilcoxon rank-sum tests, and exact *P* values are shown above each comparison. **d**, Prediction of plant nutritional traits using genetic and transcriptomic information combined with bacterial microbiome features. The bar plot shows the prediction ability of 12 mineral elements based on WGS, RNA-seq and gene expression data with or without ASV information. Results show ASV data enhance the prediction ability of 13 mineral elements. Source data

To understand how host genetic variation and gene expression regulate rhizosphere bacterial community assembly and influence plant nutritional phenotype—defined here as shoot mineral nutrient concentrations and content measured through ionomic profiling, we conducted a multi-omics analysis (Fig. 1a) integrating root transcriptomes, rhizosphere microbiomes and host genomes across 175 genetically diverse *B. napus* accessions (Supplementary Fig. 1). These accessions, representing spring, winter and semi-winter ecotypes, were grown in two contrasting field environments in China (Kaifeng (KF) and Yangling (YL)). At the flowering stage, we collected lateral roots from the longitudinal zone of the taproot for RNA sequencing (RNA-seq) and closely attached rhizosphere soil for 16S ribosomal RNA gene sequencing, with bulk soil collected as a control. A multivariate ordination analysis revealed that environmental location emerged as the largest factor shaping both gene expression and bacterial community composition, while host genotype explained more variance in root transcriptome profiles than in microbial composition (Supplementary Figs. 2 and 3). In addition, rhizosphere communities were clearly distinct from bulk soil communities, confirming a robust plant-mediated effect on bacterial assembly to the rhizosphere.

We next explored gene–microbe links by correlating highly abundant and well-characterized microbial features (that is, amplicon sequence variants (ASVs) filtered by prevalence and taxonomic resolution) with co-expressed gene modules in roots (Supplementary Datasets 2 and 3). Among 203 bacterial ASVs (Supplementary Dataset 4) with moderate to high heritability (*H*^2^ > 0.15)^35^, we identified strong associations with four root gene co-expression modules, enriched in pathways related to carbon and nitrogen metabolism, growth regulation and root development (Supplementary Dataset 5 and Supplementary Fig. 4). These functional characterizations point to a transcriptionally active root system engaged in robust growth and metabolic reprogramming, particularly in pathways related to nutrient assimilation, root development and stress adaptation—traits likely contributing to differential microbial recruitment across genotypes. Interestingly, these associations involved dominant bacterial families such as Bacillaceae, Chitinophagaceae, Comamonadaceae, Gemmatimonadaceae, Nocardioidaceae, Pseudonocardiaceae, Rhizobiaceae, Sphingomonadaceae, Vicinamibacteraceae, Xanthobacteraceae and Xanthomonadaceae, defined by high ASV richness (≥5 ASVs per family) (Supplementary Dataset 4). Their average heritability was nearly identical to that of all 203 ASVs (*H*^2^ = 0.265 versus 0.264) (Supplementary Dataset 4), indicating that dominance based on taxonomic richness does not bias heritability estimates. These findings point to transcriptionally coordinated host potentially mediating microbial recruitment.

### Host transcriptomic and rhizosphere microbial interactions predict nutrient acquisition

We then investigated whether transcriptional and microbiome variation could predict shoot nutrient levels, focusing on 12 mineral elements. Mantel’s correlations revealed significantly positive (*R* = 0.64, *P* = 0.019) association between microbial profiles, root gene expression and ionome traits—most notably for nitrogen (Fig. 1b). To quantify the relative contribution of host genetics and gene regulation to microbiome variation, we evaluated the predictive power of different omics layers—genomic markers, transcriptomic single-nucleotide polymorphisms (SNPs) and gene expression profiles—for modelling ASV abundance (Supplementary Fig. 5; Methods). Prediction models showed that gene expression alone outperformed genotype data in explaining microbiome variation and that integrating genotype data did not improve prediction of microbial abundance (Fig. 1c). This finding suggests that gene regulation captures more immediate and dynamic host responses that influence microbiome assembly, compared to static genomic variation. In other words, transcriptional activity likely reflects environmentally responsive processes such as root exudation, defence signalling and nutrient transport—factors that directly shape microbial recruitment at the root–soil interface. Building on the observation that gene expressions most strongly predict microbiome composition, we next asked whether integrating multi-omics data—including host genomic, transcriptomic and microbial features—could improve the prediction of plant nutritional traits. Integrating plant genomic, transcriptomic and rhizosphere microbial data yielded the highest average prediction accuracy for macronutrient traits—N, P, K, Ca and Mg (KF, 0.53; YL, 0.40)—while prediction performance was substantially lower for micronutrients such as B, Cu, Fe, Mn and Zn (KF, 0.30; YL, 0.10) (Fig. 1d). These results suggest that host transcriptional plasticity is more tightly linked to microbiome assembly than static genetic markers and that microbial signatures can serve as predictors of nutrient acquisition efficiency. Such integration of root transcriptional profiles with rhizosphere microbial data may advance our understanding of how host regulatory networks and microbial community structure jointly shape nutrient uptake efficiency in the field.

### Genetic and transcriptional architecture underlying host control of rhizosphere bacterial microbiota

**Fig. 2:**
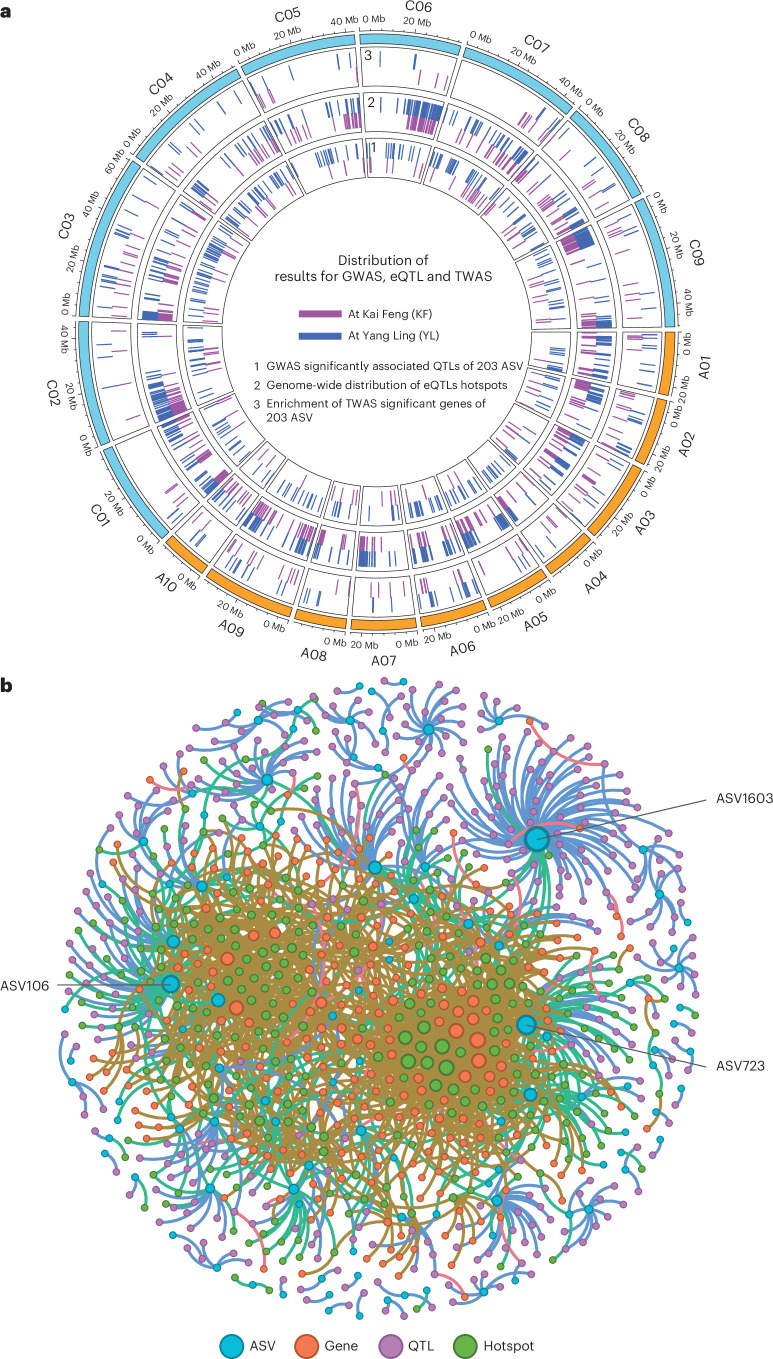
Integrative genomic and transcriptomic analyses of host–microbe associations in *B. napus.* **a**, The genomic distribution of QTLs, eQTL, and the enrichment of TWAS significant genes of all 203 ASVs. From inner to outer: 1, GWAS significantly associated QTLs of 203 ASVs; 2, eQTL hotspot detected by eGWAS; 3, TWAS significant genes identification. Purple and dark blue indicate significant correlations between GWAS, eQTL and TWAS with each ASV in KF and YL, respectively. The orange and cyan regions on the outer circles denote the rapeseed genome A and genome C, respectively. **b**, The ASV–GWAS–eGWAS–TWAS regulated network integrates results from KF and YL. Blue circles represent 106 ASVs with significant genes or QTLs, with ASV1603, ASV723 and ASV106 being the three ASVs associated with the most significant genes and QTLs. Orange circles represent 287 TWAS significant genes of ASVs. Purple circles indicate 389 GWAS significantly associated QTLs for ASVs. Green circles represent 250 eQTL hotspots. The size of each shape reflects the number of regulatory genes associated with each entity. Source data

To identify genetic loci and regulatory genes influencing rhizosphere microbiome composition, we conducted GWAS and TWAS association analyses for 203 highly heritable bacterial ASVs across two field environments (KF and YL) (Fig. 2a). Using 345,289 high-quality SNPs, GWAS revealed 169 significant SNP–microbe associations for 34 ASVs in KF, mapping to 99 independent quantitative trait loci (QTLs) (see linkage disequilibrium structure in Supplementary Fig. 6). In YL, 510 associations involving 59 ASVs were detected, corresponding to 317 QTLs. Within these intervals, we found 184 candidate genes in KF and 309 in YL (Supplementary Dataset 6). Four QTL regions were shared across both environments, and 14 ASVs—including *Sphingopyxis*-related taxa—were consistently associated with QTLs at both sites, suggesting stable host genetic effects despite environmental variability.

TWAS extended these findings by linking 36 (KF) and 46 (YL) ASVs to specific host genes based on the transcript abundance. In total, 290 unique gene–microbe associations were uncovered across both locations (Supplementary Dataset 7). Overall, the regulatory network integrating ASVs, genes and loci from GWAS, TWAS and expression quantitative trait loci (eQTL) analyses highlight key microbial taxa such as *Sphingopyxis* that are under strong host genetic regulation (Fig. 2b), suggesting a genetically encoded host capacity to select beneficial microbes.

### eQTL hotspots and long-distance regulatory variation

To further explore host regulatory architecture, we mapped eQTLs across root expressed genes and found a striking prevalence of long-distance regulation. Across both sites, >95% of eQTLs were classified as distant (>50 kb or trans-chromosomal), with only 4–5% being local (cis) (Supplementary Fig. 7 and Supplementary Dataset 8). We observed that eQTLs–expression gene (eGene) pairs located on the same chromosome exhibited significantly stronger associations than those located on different chromosomes (two-sided Wilcoxon rank sum test, *P* < 2.2 × 10^−16^; Supplementary Fig. 8), in both KF and YL locations. For the inter-chromosomal associations, distant eQTL and their target genes frequently co-localized within syntenic regions of homoeologous chromosomes between the A and C subgenomes (Supplementary Fig. 7). Based on these eQTL analyses, we identified 317 hotspots in KF and 380 in YL, characterized by enriched inter-chromosomal associations.

Despite their genomic distance, local eQTLs had a larger effect on expression variance than distant ones (two-sided Wilcoxon rank sum test, *P* < 2.2 × 10^−16^; Supplementary Fig. 9), for both KF and YL locations. Still, the widespread presence of distal eQTLs—regulating 11,978 and 11,976 eGenes in KF and YL, respectively—suggests that complex, trans-acting networks contribute to genotype-dependent expression patterns linked to microbiome assembly. A substantial fraction of eGenes (~30%) were regulated by only one or two eQTLs, implying discrete regulatory control over specific genes (Supplementary Fig. 10). Altogether, these findings illustrate a multi-layered genetic architecture—comprising local and distal regulation—through which host plants shape their rhizosphere microbiomes. Importantly, this architecture also underlies variation in nutrient uptake, highlighting tractable targets for breeding nutrient-efficient and microbiome-responsive crops.

### Multi-omics integration reveals host genetic of *Sphingopyxis* in the rhizosphere

To identify bacterial taxa under strong host genetics and transcriptional control, we constructed a comprehensive multi-omics regulatory network by integrating GWAS, TWAS and eQTL results across 203 heritable ASVs (Supplementary Dataset 9). The resulting network, compiled from both KF and YL datasets, contained 106 ASVs, 389 ASV-associated QTLs, 287 TWAS-significant genes and 250 eQTL hotspots, highlighting that the genetic and gene regulatory effect on host–microbe association is extremely complex in *B. napus* (Supplementary Dataset 9). Phylogenetic analysis of all 203 ASVs further revealed deep diversity and taxonomic structure (Supplementary Fig. 11). To pinpoint key microbial features, we applied stepwise regression to link ASVs with gene expression patterns across all 17,006 genes. This analysis identified the top ASVs most frequently associated with gene expression variation in KF (for example, ASV407, ASV23, ASV460) and YL (for example, ASV5542, ASV205, ASV344). Phylogenetic mapping of these top ASVs revealed a single overlapping clade across both environments: the genus *Sphingopyxis*, represented by ASV407, ASV723, ASV3382 and ASV5542 (Supplementary Fig. 11). This convergence led us to focus on *Sphingopyxis* as a candidate taxon under robust host genetic regulation.

### Colonization and function of *Sphingopyxis* associates with host expressed genes under stress

We further examined the specific regulatory architecture of *Sphingopyxis* abundance by constructing a taxon-specific regulatory network using expression genome-wide association study (eGWAS), GWAS and TWAS. This *Sphingopyxis*-focused network comprised 416 QTLs, 290 TWAS-associated genes and 697 eQTL hotspots, emphasizing the complexity of its host-mediated assembly process (Supplementary Dataset 9). To this end, our integrative analyses revealed interactions between host nitrogen-related metabolism and the rhizosphere microbiota. ASV723 was associated with host variates enriched in Gene Ontology (GO) terms ‘organonitrogen compound metabolic process’ (GO 1901564, adjusted *P* = 0.00652) and ‘nitrogen compound metabolic process’ (GO 0006807, adjusted *P* = 0.00766) (Supplementary Fig. 12), suggesting that this taxon may involve nitrogen homeostasis by modulating specific metabolic pathways in rapeseed. Among the genes associated with ASV723 interactions, six were functionally annotated and implicated in carbon and amino acid metabolism. For example, BnaA01g21040D encodes a casein kinase II, alpha chain 2 (CK2), whose activity is known to affect auxin-dependent processes, particularly auxin transport. BnaA01g23120D encodes a protein with serine/threonine kinase activity and has been reported to be involved in abscisic acid signal transduction. BnaA02g29390D belongs to the cytochrome P450 superfamily, which is involved in glucosinolate metabolism. BnaA05g05760D is an orthologue of *Arabidopsis thaliana* gene P5CS1, encoding the delta1-pyrroline-5-carboxylate synthase, the enzyme responsible for the rate-limiting step in proline biosynthesis. BnaC07g46780D is orthologous to *A. thaliana* AT4G37800, a member of the endotransglucosylase/hydrolase gene family, involved in carbon metabolism (GO 0005975) and cell wall organization (GO 0005618). BnaC09g12040D encodes a transcription factor from the Dof zinc finger family. These six ASV723-associated genes are functionally linked to carbon and amino acid metabolism, highlighting potential roles in hormone signalling, stress responses and cell wall organization in rapeseed.

### *Sphingopyxis* enhances root development via auxin biosynthesis

**Fig. 3:**
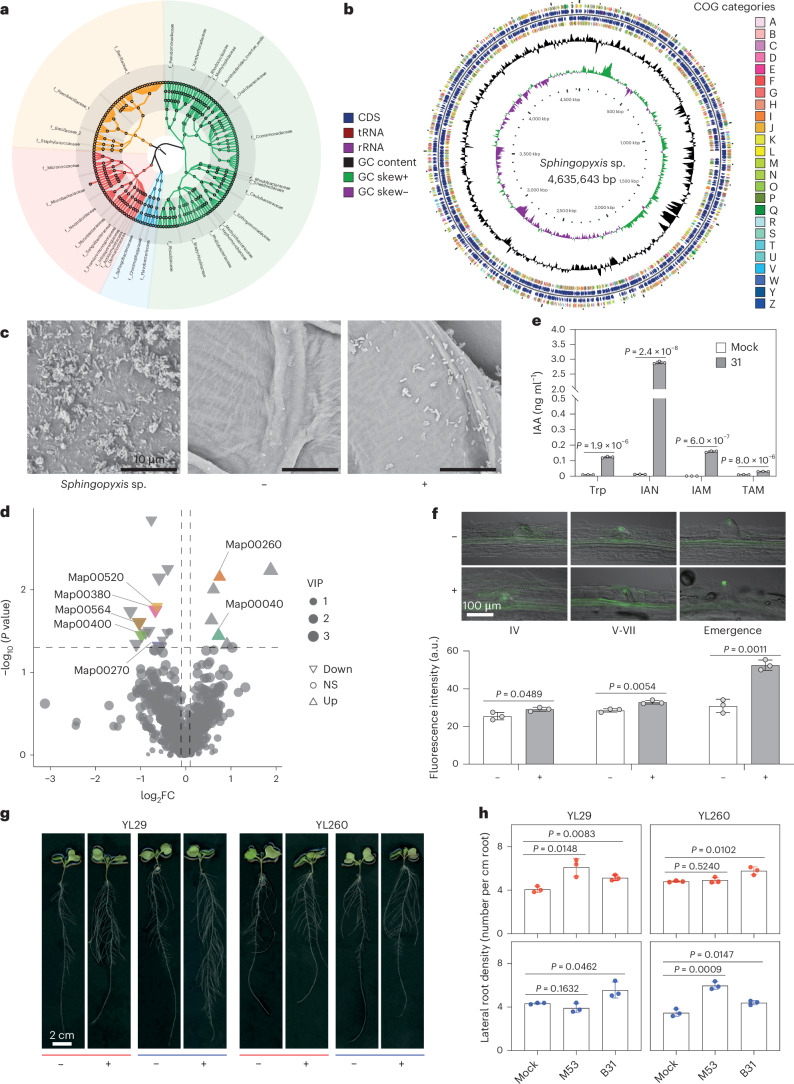
Identification of a novel bacterial taxon *Sphingopyxis* involved in auxin-mediated lateral root formation in *B. napus.* **a**, High-throughput bacterial cultivation and identification from the *B. napus* root system. Different coloured clades indicate distinct bacterial orders. **b**, Whole genome assembly of the novel bacterial genus *Sphingopyxis*. Circular genome map of the *Sphingopyxis* isolate derived from the rapeseed root. The genome was assembled using a hybrid approach combining Nanopore and Illumina sequencing, resulting in a total size of 4,635,643 bp. The circular plot shows (from outer to inner rings): (1) protein-coding genes on the forward strand, coloured by COG functional categories; (2) protein-coding genes on the reverse strand; (3) tRNA and rRNA genes; (4) GC content; and (5) GC skew (green and purple indicate positive and negative skew, respectively). This map provides an overview of genomic features and gene function assignments, including enriched categories relevant to amino acid transport, nitrogen metabolism and hormone biosynthesis. **c**, Scanning electron microscopy images of the root surface grown without (middle) or with *Sphingopyxis* isolate 31 (right). Scale bars, 10 μm. **d**, Untargeted metabolite profiling putatively identified the differential metabolites involved in diverse biological pathways after *Sphingopyxis* inoculation (isolate 31). Log-transformed fold changes and adjusted *P* values are shown. Upward and downward triangles indicate metabolites with significantly increased or decreased accumulation following *Sphingopyxis* inoculation, respectively. Statistical significance was determined using a two-sided Student’s *t*-test with Benjamini–Hochberg FDR correction for multiple comparisons. Grey circles represent non-significant changes (NS, adjusted *P* ≥ 0.05). Significant metabolites annotated to KEGG pathways are labelled accordingly. Map00040 (pentose and glucuronate interconversions), Map00260 (glycine, serine and threonine metabolism), Map00270 (cysteine and methionine metabolism), Map00380 (tryptophan metabolism), Map00400 (phenylalanine, tyrosine and tryptophan biosynthesis), Map00520 (amino sugar and nucleotide sugar metabolism), Map00564 (glycerophospholipid metabolism). FC, fold change. VIP, variable importance in projection. **e**, The concentration of IAA in mock supernatant or isolate 31 supernatant co-cultured with or without Trp, IAN, IAM and TAM. Statistical significance was assessed using a two-sided Student’s *t*-test, and exact *P* values are shown above each comparison. Data are shown as means ± s.d. (*n* = 3 biological independent replicates). **f**, DR5::GFP staining of *Arabidopsis* primary root after inoculation with *Sphingopyxis* (isolate 31). The lateral root primordium was classified based on different developmental stages. Quantification of DR5::GFP fluorescence intensity (bottom) is shown as mean ± s.d. (*n* = 3 biologically independent roots). Statistical significance was assessed using a two-sided paired Student’s *t*-test, and exact *P* values are shown above each comparison. **g**, Growth performance of *B. napus* genotypes YL29 and YL260 under high (100%, shown in red) and low (50%, shown in blue) nitrogen conditions with or without *Sphingopyxis* inoculation (isolate 31) in the agar system. Scale bar, 2 cm. **h**, Lateral root density of *B. napus* genotypes YL29 and YL260 under high (100%, shown in red) and low (50%, shown in blue) nitrogen conditions with or without *Sphingopyxis* inoculation (isolate 31) in the soil pot system. Another bacterial isolate M53 that belongs to *Sphingopyxis* was derived from *Medicago* root. The significances were controlled by two-sided paired Student’s *t*-test, and exact *P* values are shown above each comparison. *n* = 3 biologically independent replicates. Source data

To evaluate the functional role of *Sphingopyxis*, we performed high-throughput bacterial cultivation^36^ using root samples from rapeseed varieties YL29 (semi-winter ecotype) and YL260 (winter ecotype), which had shown relatively high *Sphingopyxis* abundance based on our sequencing data. This made them suitable candidates for isolating the bacterium for downstream validation in soil pots. Overall, this approach yielded root-derived colony-forming units (c.f.u.) representing 4 bacterial phyla, and 32 bacterial families associated with the rapeseed root microbiota (Fig. 3a). We next mapped the 16S rRNA gene sequences of the cultivated isolates to ASVs classified as *Sphingopyxis* from our large-scale field experiments, selecting only those with >97% sequence similarity for subsequent soil inoculation experiments. In particular, for bacterial isolate 29–6000–31, classified as *Sphingopyxis*, we successfully assembled a complete genome of 4,635,643 bp (Fig. 3b). Comparative analysis of clusters of orthologous groups (COG) in the *Sphingopyxis* genome identified 3,498 protein-coding genes, with significant (adjusted *P* < 0.05) enrichment into 222 biological pathways, particularly those related to amino acid transport and metabolism (Supplementary Dataset 10). Finally, we inoculated rapeseed grown in soil pots under low and high nitrogen conditions with *Sphingopyxis* isolate 29–6000–31 and confirmed its successful colonization of the root surface (Fig. 3c). We further profiled the root metabolome features using untargeted metabolomics and identified that inoculation of *Sphingopyxis* significantly altered the accumulation of diverse metabolites putatively identified as amino acid derivatives, flavonoids, phenolic acids and sugars (Fig. 3d, Supplementary Dataset 11 and Supplementary Fig. 13a), suggesting a broad impact on plant metabolic pathways related to root development and nutrient signalling. Auxin-related metabolites such as indole-3-acetic acid (IAA) and its biosynthetic precursors were also putatively identified under low nitrogen conditions, suggesting that *Sphingopyxis* may influence root development via modulation of auxin pathways (Supplementary Fig. 13). Specifically, high-performance liquid chromatography coupled to tandem mass spectrometry (HPLC–MS/MS) analysis showed that *Sphingopyxis* produced IAA when supplied with different tryptophan-derived substances—including Trp, indole-3-acetonitrile (IAN), indole-3-acetamide (IAM), and tryptamine (TAM)— indicating its capacity to use multiple IAA biosynthesis pathways (Fig. 3e, Supplementary Datasets 12 and 13 and Supplementary Fig. 13). Auxin reporter imaging using DR5::GFP, a synthetic auxin-responsive reporter construct in which a multimerized auxin response element (DR5) drives expression of green fluorescent protein (GFP), showed that inoculation of *Sphingopyxis* modulated auxin transport from the root tip (Supplementary Fig. 14) to the lateral root (Fig. 3f) and significantly (*P* = 1.0 × 10^−8^, analysis of variance, Tukey honestly significant difference) promoted lateral root density of two cultivars (YL29 and YL260) under both nitrogen conditions (Fig. 3g,h). We applied another *Sphingopyxis* isolate (M53) recovered from *Medicago*, and this isolate also partially showed the promotion effect on the increase of lateral root density (Fig. 3g,h). Thus, this novel bacterium *Sphingopyxis* might provide consistently promotive effect on root development by mediating the auxin biosynthesis and transport-related processes in plants.

### *Sphingopyxis* supports nitrogen uptake and interacts with host genes under stress

**Fig. 4:**
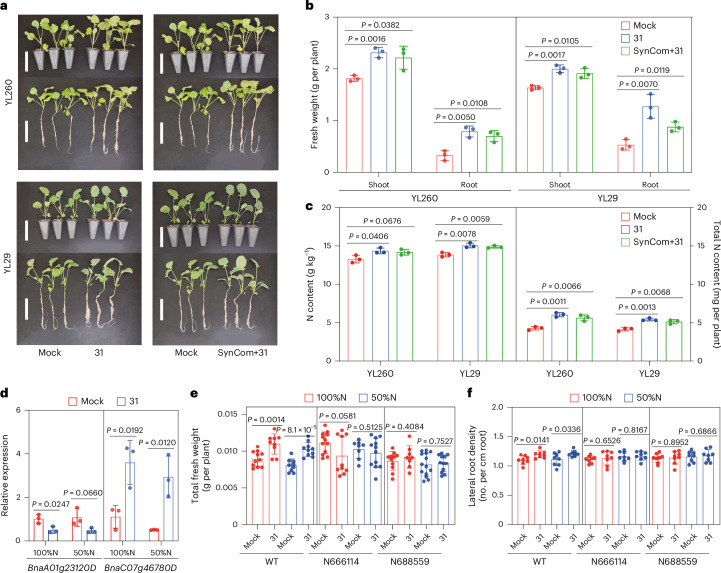
*Sphingopyxis* driven rapeseed performance depends on specific plant genes. **a**, Growth of rapeseed genotypes with single inoculation using isolate 31 or with a SynCom including isolate 31 under low nitrogen (50% full) conditions. Scale bars, 10 cm. **b**, Fresh weight of rapeseed after inoculation with isolate 31 or SynCom containing isolate 31. *n* = 3 biologically independent replicates. **c**, Shoot nitrogen concentration and content after inoculation with single isolate or SynCom with isolate 31. *n* = 3 biologically independent replicates. **d**, Real-time PCR quantification of relative expression of two rapeseed genes (*BnaA01g23120D*, *BnaC07g46780D*). *n* = 3 biologically independent replicates. **e**, Total fresh weight of T-DNA inserted *Arabidopsis* mutants N666114 and N688559, which are encoded by orthologous genes *BnaA01g23120D* and *BnaC07g46780D*, respectively. *n* = 10 biologically independent replicates. **f**, Lateral root density of *Arabidopsis* mutants N666114 and N688559. *n* = 8 biologically independent replicates. In **b**–**f**, statistical significance was evaluated using a two-sided Student’s *t*-test, with exact *P* values displayed above each comparison. Bars represent mean ± s.d. Source data

Beyond root development, *Sphingopyxis* inoculation improved shoot biomass (Fig. 3g) and nitrogen accumulation (Fig. 3h), particularly under low nitrogen conditions. Although *Sphingopyxis* does not contribute to the nitrogen fixation, as confirmed by growth failure on nitrogen-free medium (Supplementary Fig. 15), it likely facilitates nitrogen uptake through indirect mechanisms. To investigate its interaction with other bacterial members (Supplementary Dataset 14), we conducted a Synthetic Community (SynCom) experiment with and without *Sphingopyxis*. Whether applied alone or in combination, *Sphingopyxis* significantly enhanced shoot and root biomass (Fig. 4a,b) and nitrogen accumulation (Fig. 4c), especially under nitrogen-poor conditions. These results suggest that *Sphingopyxis* can stably promote rapeseed growth in nitrogen-limited soil, likely through cooperative interactions with other bacterial taxa.

**Fig. 5:**
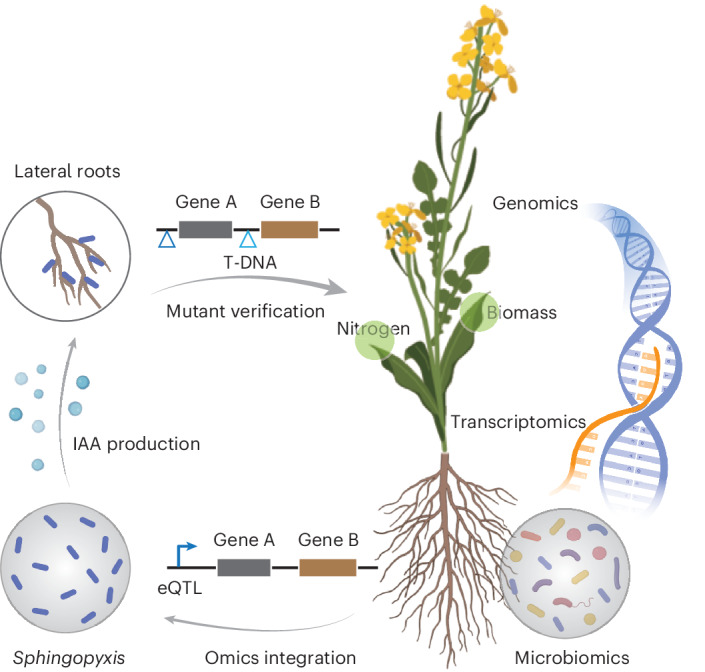
A schematic model depicting the potential of omics prediction on identification of causal plant–microbial interactions in *B. napus.* This model integrates multi-omics data, including genomic, transcriptomic and microbiome information, to predict key microbial taxa and plant genes that drive important plant traits such as root development, biomass production and nitrogen acquisition. The model illustrates how genomic data (GWAS and eQTL) and transcriptomic data (TWAS) interact with microbial community features (ASVs) to predict plant microbial interactions. Functional verifications, such as auxin biochemical assays, microbial colonization and plant mutant studies, validate the predicted interactions. The results highlight the importance of host–microbe co-regulation for optimizing crop performance under varying environmental conditions. Figure created with BioRender.com.

To explore whether the expression of plant genes is associated with taxon *Sphingopyxis*, we performed real-time PCR to quantify the relative expression with and without *Sphingopyxis* inoculation. As shown in Fig. 4d and Supplementary Fig. 16, five out of six candidate genes were significantly upregulated following inoculation, especially under low nitrogen conditions. In addition, we identified new *A. thaliana* mutants with altered expression of these candidate genes. Among these, the orthologues of BnaA01g23120D and BnaC07g46780D showed consistent expression changes in comparison to the wild type. In particular, *Sphingopyxis* inoculation dramatically increased total fresh weight and lateral root number in the wild-type plants, but this growth-promoting effect was abolished in mutants of either gene (Fig. 4e,f). Taken together, these results suggest that expression of BnaA01g23120D and BnaC07g46780D is potentially linked to the inoculation and colonization of *Sphingopyxis*. Overall, this study highlights the power of multi-omics approaches to uncover host–microbe associations and identify novel bacterium that may contribute to systemic growth promotion and nutrition uptake in rapeseed (Fig. 5).

## Discussion

Plants have evolved specialized rhizosphere systems that not only facilitate nutrient acquisition but also host diverse microbial communities shaped by host metabolic and generic components^37^. Increasing evidence indicates that plant–microbiome associations are under genetic control across various plant species^17–23^ and show measurable heritability^33,35^. However, the gene regulatory mechanisms underlying microbiome assembly remain poorly understood in crops. This knowledge gap stems from two key challenges. First, it is unclear to what extent non-leguminous plants can actively regulate gene expression to shape microbial recruitment, unlike legumes where transcriptional control of nodulation is well established^38^. While some rhizobia species can colonize cereal crops^39^, they fail to provide efficient nitrogen fixation without sustained nitrogenase activity^40^. Second, there is a lack of integrative, population-scale studies that connect host genome, transcriptome and microbiome variation, particularly under field conditions.

In this Article, we present the first comprehensive multi-omics analysis of plant–microbe interactions in the *B. napus* crop, linking host genetic variation and gene expression to microbial community composition and plant nutritional traits in the field. Our study adhered to rigorous standards and unified framework, integrating a large set of paired datasets including 1,341 host SNP array, root transcriptome and rhizosphere 16S rRNA bacterial microbiome data, alongside 12 root ionome traits measured across two distinct environments. Through integrative network association and covariation analyses of the host genome, transcriptome and inhabited bacterial microbiome, we identified key molecular components and pathways potentially linked to heritable bacterial taxonomic ranks associated with root nitrogen status (Fig. 1). We emphasize that plants can play an active role in preferentially selecting microbes that enhance nitrogen nutrition, a critical process due to nitrogen’s abundance in the atmosphere and its essential role in plant life cycle. Leveraging the power of population genomic and transcriptomics, we demonstrate that the rhizosphere bacterial microbiome substantially improves the predictability of plant nutritional traits, particularly when integrated with host transcriptomic data. Furthermore, we identify significant genome-wide associations between bacterial characteristics in the rhizosphere and root nitrogen-related genes, along with other host genetic factors. By quantifying the total contribution of host genetic loci to bacterial microbiome variation across chromosomes, we provide insights into the genetic regulation of host–microbe interactions. The integration of local and distal eQTLs revealed a complex regulatory architecture in which gene expression variation—especially from distal, trans-regulatory regions—plays a major role in shaping microbiome composition (Fig. 2). This work provides a valuable resource for exploring regulatory networks and host control points in governing crop-associated microbial communities.

In particular, we identified a novel bacterial taxon, *Sphingopyxis*, whose abundance variation is strongly associated with plant gene expression regulated by eQTL hotspots, suggesting a genetically controlled recruitment mechanism (Fig. 3). High-throughput cultivation and whole-genome sequencing provided the complete genomic information for *Sphingopyxis*, revealing a broad range of functional characteristics, including nitrogen and hormone metabolism. Targeted HPLC–MS/MS analysis confirmed that *Sphingopyxis* can produce IAA in vitro when supplemented with tryptophan-derived metabolites, and untargeted metabolomic profiling of inoculated plants putatively identified compounds associated with tryptophan metabolism. While these findings suggest a potential link between *Sphingopyxis*-mediated auxin production and modulation of lateral root development, further genetic or isotope-based evidence is needed to directly demonstrate in situ IAA production and its causal role in shaping root phenotypes. This finding aligns with known mechanisms of root-microbiota-mediated hormonal modulation in *Arabidopsis*^5,41,42^. We finally demonstrated that a specific *Sphingopyxis* strain facilitates root development and nitrogen acquisition from bacterial isolates derived from both rapeseed and *Medicago* under different conditions (Fig. 3). Indeed, root development and differentiation could coordinate microbiota assembly and plant ionomic homeostasis^3,43^. Such specific microbe-driven regulation of root development could offer a notable growth advantage for crop plants under resource-limited conditions^31,32^. Our results confirm that host genetic variation impacts root development and nitrogen uptake, with these traits contributing to improved host performance^33^. These beneficial plant–microbe interactions are regulated by plant genes involved in nutrient transport^31^ and root development^33^, which may facilitate breeding efforts aimed at improving crop symbioses and adaptive root phenotypes. Our quantitative PCR and mutant analyses suggest that *Sphingopyxis*-responsive genes, such as *BnaA01g23120D* and *BnaC07g46780D*, play a key role in mediating microbial effects on root growth and nitrogen acquisition, as mutations in their *Arabidopsis* orthologues disrupted the bacterium’s growth-promoting effect (Fig. 4). These results highlight a causal link between host gene expression and the establishment of beneficial plant–microbe interactions, thus reinforcing the concept that host genes regulating nutrient signalling and root development are central to shaping beneficial microbial partnerships. These findings provide a critical step toward closing the knowledge gap between host genetics, gene regulation and biotic interactions with the rhizosphere. The functional interactions we observed will offer strong evidence of causality that could be used to translate host–microbe associations into enhanced crop resilience under field conditions.

In the context of this study, we define plant fitness as the integrative outcome of genetic, transcriptional, and microbial interactions that contribute to optimal nutrient acquisition—particularly nitrogen—and sustain biomass production in the field. Plant health is characterized by a well-regulated ionomic profile, stable growth under nutrient-limited conditions and responsiveness to growth-promoting rhizobacteria. Our predictive modelling shows that nitrogen content, a key fitness proxy, can be accurately predicted by combining root transcriptomic and rhizosphere microbial features. Furthermore, the identification of *Sphingopyxis* as a keystone taxon that modulates auxin biosynthesis, root development and nitrogen accumulation directly links microbial association patterns with measurable fitness-related traits. These results suggest that host–microbiome interactions not only are reflective of plant health status but also can be harnessed as predictive indicators for breeding nutrient-efficient and resilient rapeseed varieties.

Despite these insights, our study has some limitations. Although our primary analyses were conducted across all genotypes, initial subgroup comparisons suggest that ecotype-specific variation—particularly between spring and winter lines—may influence certain microbial associations and transcriptomic patterns. Further targeted analyses with balanced designs will be needed to resolve ecotype-driven omics signatures and their contribution to microbiome assembly. While our study relies on 16S rRNA amplicon sequencing, which limits species-level resolution and functional inference, the robust genotype–microbiome associations observed at the genus level remain highly informative. Future work incorporating full-length 16S rRNA gene sequencing, metagenomics or culture-based approaches will be essential to refine taxonomic resolution and explore microbial functions more precisely. Future research should not only focus on bacteria but also consider fungi and bacterivore protists to validate and enhance the understanding of beneficial microbiomes by using metagenomic and metatranscriptomic approaches in crops. In addition, further studies are needed to explore the complex interaction effects of plant genotypes and growth stages on the assembly and function of specific microbial taxa. Given the limited number of *Sphingopyxis* strains in this study, a promising avenue for future research is to investigate the functional diversity of isolates within this taxon, sourced from diverse soil origins and plant species, using a microbial pan-genomics approach. Although glucosinolates were not the central focus of this study, our TWAS results suggest that genes involved in glucosinolate metabolism, such as *BnaA02g29390D*, may influence specific microbial associations. Future studies incorporating glucosinolate profiling and functional validation will be important to further elucidate their potential role in shaping root-associated microbiota, including taxa like *Sphingopyxis*. Despite these limitations, our study provides valuable insights into the stepwise process of microbial assembly, from host genome to transcriptome, and ultimately to the development of population-based metabolomics. This work lays the foundation for future efforts to translate multi-omics insights into actionable strategies for breeding nutrient-efficient and microbiome-responsive crops.

## Methods

### Plant materials and field preparation

A panel of 300 *B. napus* accessions, representing global diversity, was sown in the field^44^. This panel consists of 187 winter, 55 semi-winter and 58 spring ecotypes, classified based on genetic information and field observations (Supplementary Fig. 1). Detailed sample sizes and ecotype information for each geographical origin are provided in Supplementary Dataset 1. In September 2019, all accessions were sown in a randomized complete design (30 cm row spacing, 15 cm plant spacing), with three biological replications corresponding to three rows, across two field locations: KF in China (34°47′ N, 114°18′ E) and YL (34°17′ N, 108°03′ E). The KF site represents a typical cropping system in the North China Plain, characterized by a winter rapeseed–summer maize rotation, while the YL site reflects an arid agricultural system. Before planting, the basic physical and chemical properties of the soil at both sites were analysed for the top 30 cm soil (Supplementary Table 1), alongside climatic parameters including temperature and precipitation, to characterize the environmental conditions that may influence rapeseed growth and flowering performance. To ensure proper seed germination, both plots were sufficiently irrigated to achieve optimal soil moisture at least 1 month before sowing. In addition, plant material from the previous season was removed from the fields. Fertilization was applied uniformly across both plots and incorporated into the surface soil by rotary tillage before sowing. The fertilization regime included 75 kg P ha^−1^ (calcium superphosphate), 75 kg N ha^−1^ (urea) and 75 kg K ha^−1^ (potassium sulfate). The planting density was standardized at approximately 165,500 plants per hectare, with rows spaced 40 cm apart. No herbicides or additional irrigation was applied during the growth period; plants relied solely on natural rainfall following the initial pre-sowing irrigation.

### Rhizosphere and root collection at two field locations

Due to environmental variation and soil differences, only 175 genotypes successfully grew and were collected at both locations, although a total of 300 rapeseed genotypes were sown at both field sites. These 175 genotypes were used for downstream nutrient profiling and multi-omics analysis to ensure consistency in cross-site comparisons. Rhizosphere samples were collected from 150-day-old rapeseed plants at the flowering stage at both field locations. In detail, the entire root systems were carefully excavated from the soil, with the sampling depth ranging from 30 cm to 60 cm, depending on the distribution of the main roots in the soil profile. The soil tightly adhering to the roots was defined as the rhizosphere, after rigorously shaking off any loosely attached soil particles. As rapeseed typically has a single main root with highly branched lateral roots, we specifically dissected the lateral roots to ensure consistent sampling for accurate comparison among genotypes. The soil closely attached to the lateral roots was carefully removed using a brush without damaging the roots, following the method previously described^32^, and defined as rhizosphere. Rhizosphere samples were collected from two representative plants randomly selected from each plot, with the two samples pooled together for each genotype. These fresh rhizosphere samples were sieved through a 1 mm mesh to remove visible root fragments and then stored at −80 °C for subsequent DNA extraction. To ensure high-quality RNA samples from the roots, the same sampling procedure was repeated for another two plants. The soil adhered to the roots was immediately washed off using tap water, followed by two rinses with sterile distilled water to minimize potential contamination. Lateral roots were carefully separated using sterilized forceps and collected as described during the rhizosphere collection. The washed lateral roots were rinsed twice in sterilized water to eliminate any potential contaminations, dried using clean tissue, and frozen in 15 ml Falcon tubes with liquid nitrogen before being stored at −80 °C for RNA extraction. Bulk soil samples were also collected from the middle of the rows, at a depth of 30 cm, serving as the control. These bulk soil samples were thoroughly mixed, and a representative proportion was taken for downstream analysis.

### Shoot collection and ionome profiling

The complete aboveground parts (mixture of stem and leaves) of rapeseed plants from all 175 genotypes were gathered on the day of collection at both KF and YL fields. These plant materials were first heat treated at 105 °C for 30 min and then dried at 70 °C to a constant weight. The dried plant parts were weighed to determine the shoot dry biomass. Subsequently, the dried samples were initially ground coarsely using a laboratory crusher (BF-10, Hebei Beichen Technology), followed by fine grinding to a powder using a ball milling machine (Multifunctional ball mill QM100S, Beijing Wuzhou Dingchuang Technology). Ionome profiling involved 12 mineral nutrients, as previously described^32^. In brief, 2–5 mg of finely ground plant powder was used to measure total nitrogen concentration via an elemental analyser (Isotope ratio mass spectrometer, Vario PYRO Cube-IsoPrime 100, Elementar-Isoprime). The data were processed into peak areas using the Callidus software (version 1.1), which provided quantitative results with reference material. The same plant material was also analysed for 11 additional mineral nutrients using an inductively coupled plasma optical emission spectrometer machine (19A07591, Agilent Technology).

### Genomic DNA extraction and bacterial community profiling in the rhizosphere

Approximately 250 mg soil was used for genomic DNA extraction for a total of 1,341 rhizosphere samples, derived from 175 rapeseed genotypes with three biological replications at two field locations with additional 72 bulk soil samples as control, using the E.Z.N.A. Soil DNA Kit (Omega Bio-tek) according to the manufacturer’s protocol. All DNA samples were assessed for quality, and DNA concentrations were quantified using a NanoDrop 2000 spectrophotometer (Thermo Fisher Scientific). To profile the bacterial community, the hypervariable V3–V4 region of the bacterial 16S rRNA gene was amplified using the primers 338F (5′-ACTCCTACGGGAGGCAGCAG-3′) and 806R (5′-GGACTACHVGGGTWTCTAAT-3′). PCR amplification was carried out under the following conditions: an initial denaturation at 95 °C for 30 s, followed by 27 cycles of denaturation at 95 °C for 30 s, annealing at 55 °C for 30 s and extension at 72 °C for 45 s. A final extension step was performed at 72 °C for 45 s, with samples stored at 4 °C. The PCR reaction mixture consisted of 4 μl of 5× TransStart FastPfu buffer, 2 μl of 2.5 mM deoxynucleoside triphosphates, 0.8 μl of each 5 μM oligonucleotide primer, 0.4 μl TransStart FastPfu DNA Polymerase, 10 ng extracted DNA and double-distilled H_2_O to a final volume of 20 μl. Agarose gel electrophoresis was used to verify the size of amplicons, and successful amplicons were subjected to paired-end sequencing on the Illumina MiSeq PE300 platform.

After demultiplexing, the resulting sequences were first quality filtered using fastp (v0.19.6)^32,45^, and high-quality reads were then merged using FLASH (v1.2.11)^46^. The qualified sequences were then de-noised using the DADA2^47^ plugin in the Qiime2 pipeline (v2020.2)^48^, with default parameters, to achieve single-nucleotide resolution based on error profiles within the samples. The denoised sequences, referred to as ASVs, were taxonomically classified using the sklearn-based Naive Bayes classifier implemented in Qiime2 and the SILVA 16S rRNA database (v138). Based on the ASV data, rarefaction curves and the Shannon index for alpha diversity were calculated using Mothur (v1.30.1). The similarity between bacterial communities across different samples was assessed by calculating the Bray–Curtis dissimilarity matrix and then visualized using principal coordinate analysis via the R Vegan (v2.4-3) package. To assess the statistical significance and the percentage of variation explained by the treatment, the permutational multivariate analysis of variance (*n* = 999) test was conducted using R Vegan (v2.4-3) package.

### Root RNA sequencing and gene expression analysis

RNA was extracted from root tissue using TRIzol reagent according to the manufacturer’s instructions (Invitrogen), and genomic DNA was removed using DNase I (TaKara). RNA quality was assessed by the Agilent 2100 Bioanalyser, and only high-quality RNA samples with RNA integrity number greater than 8.0 were used for library construction. RNA-seq libraries were prepared using the TruSeqTM RNA Sample Preparation Kit (Illumina) with 1 μg of total RNA. Briefly, messenger RNA was isolated via polyA selection using oligo(dT) beads and then fragmented with a fragmentation buffer. Subsequently, double-stranded complementary DNA was synthesized using the SuperScript Double-Stranded cDNA Synthesis Kit (Invitrogen) with random hexamer primers (Illumina). The synthesized cDNA was then processed for end repair, phosphorylation and ‘A’ base addition, as outlined in Illumina’s library construction protocol. Libraries were selected for cDNA fragments of approximately 300 bp using 2% low-range ultra agarose gel electrophoresis, followed by PCR amplification for 15 cycles with Phusion DNA polymerase (NEB). After quantification using the TBS380 fluorometer, paired-end RNA-seq libraries were sequenced on the Illumina NovaSeq 6000 sequencer (2 × 150 bp read length).

The raw paired-end reads were trimmed and quality controlled using fastp (version 0.19.5, https://github.com/OpenGene/fastp) with default parameters^45^. The clean reads were aligned to the *B. napus* reference genome (available at https://www.genoscope.cns.fr/brassicanapus/data/) in orientation mode using HISAT2 (version 2.1.0, http://ccb.jhu.edu/software/hisat2/index.shtml)^49^. Gene expression levels were quantified using the transcripts per million reads method. The RSEM (version 1.3.1, http://deweylab.biostat.wisc.edu/rsem/) software^50^ was used to calculate gene expression from the aligned reads.

### Weighted correlation network analysis and correlation with phenotypic traits

To explore the relationships between gene expression and bacterial community composition, we applied weighted correlation network analysis (WGCNA), an unbiased, data-driven approach to identify biologically relevant associations between gene expression and bacterial taxonomic ranks^51^. WGCNA (v1.72.1) in R was used to identify distinct gene expression modules, each representing different patterns of correlation among genotypes across the two field conditions. We filtered and normalized the gene expression table to construct a robust network of co-expressed gene modules. The soft thresholding power (*β*) was chosen by default to calculate the adjacent matrix. This matrix was then transformed into a topological overlap matrix (TOM), and the corresponding dissimilarity matrix (dissTOM) was calculated as ‘1 − TOM’ to minimize the influence of noise. We then used dissTOM as the distance measure to detect co-expressed gene modules, with a minimum module size set to 30 ASVs. To further characterize these modules, we calculated the ‘eigengene’ for each module, which was then correlated with various ionomic traits and bacterial ASVs. Modules with a Spearman correlation coefficient >0.1 and a *P* < 0.05 were considered significantly associated with the traits. Finally, significant modules were visualized using Cytoscape (v3.8.0) for network representation.

### Heritability estimation for bacterial ASVs

We estimated the broad-sense heritability (*H*^2^) of bacterial ASVs in the rhizosphere; we first filtered for robust ASVs by retaining only those with a total read count of ≥10,000 reads and detected in at least 80% of samples^33,35^. Relative abundances of the ASVs were then normalized and log-transformed to reduce compositional bias and approximate a normal distribution. For each ASV, we applied a linear mixed model separately within each field location (KF and YL) to partition the observed variance:

$${y}_{{ijk}}=\mu +{g}_{i}+{r}_{j}+{b}_{{jk}}+{e}_{{ijk}},$$

where ${y}_{{ijk}}$ is the log-transformed abundance of a given ASV for genotype *i*, replicate *j* and block *k*; $\mu$ is the overall mean; ${g}_{i}$ is the random effect of the *i*th genotype, ${r}_{j}$ is the random effect of the *j*th replicate; ${b}_{{jk}}$ is the random effect of the *k*th block nested within the *j*th replicate, and ${e}_{{ijk}}$ is the residual error. All effects except the general mean were assumed to be random and were modelled to follow independent normal distribution.

The heritability was calculated using the following formula:

$${H}^{2}=\frac{{\sigma }_{g}^{2}}{{\sigma }_{g}^{2}+{\sigma }_{e}^{2}/R},$$

where ${\sigma }_{g}^{2}$ and ${\sigma }_{e}^{2}$ are the estimated genotypic and residual variance, and *R* is the number of replications. This metric quantifies the proportion of total variation in ASV abundance attributable to host genetic variation, thus identifying ASVs potentially influenced by plant genotype.

The best linear unbiased estimations (BLUEs) of all genotypes for each ASV at each location were obtained by fitting the model again, assuming the general mean and genotypic effects were fixed, while all other effects were treated as random. This approach allows for accurate estimation of genotype effects while accounting for the random variation introduced by other factors (such as replicates and blocks). All linear mixed models were fitted using the software ASReml-R 4.0 (ref. ^52^).

### Linkage disequilibrium

Linkage disequilibrium decay with physical distance between pairwise SNPs was calculated within a 500 kb sliding window and visualized using PopLDdecay software (v3.41)^53^ with default parameters. This analysis was performed separately for whole genome sequencing (WGS) SNPs and transcriptome sequencing (RNA-seq) SNPs at the KF and YL field locations, respectively. Linkage disequilibrium decay was quantified at the distance at which the linkage disequilibrium value decreased to 0.1, with the results showing a decay at approximately 1.5 kb for WGS SNPs and 50 kb RNA-seq SNPs (Supplementary Fig. 13). For the purpose of QTL region identification, a 50 kb window was used in both GWAS and eQTL analyses.

### GWAS linking host genotype to abundance of heritable bacterial ASVs

To assess the association between genomic regions and the rhizosphere bacterial microbiomes, the abundance of bacterial ASVs was treated as a quantitative trait. For genotypic data, a total of 345,289 WGS SNPs, after imputation using Beagle v5.2 (ref. ^54^), were retained. These SNPs had a minor allele frequency (MAF) ≥ 0.05 and a genotype heterozygosity rate ≤95% across the entire population. A standard single variant (SNP)-based ‘*Q* + *K*’ GWAS analysis was performed using a mixed linear model^55^ to apply associations for all 203 ASVs. A marker-derived kinship matrix was used to correct for multiple levels of relatedness and control for polygenic background effects. The model was formulated as follows:

$${\mathbf{y}}=X{\mathbf{\upbeta}} +{\mathbf{m}}{a}+g+e,$$

where ${\mathbf{y}}$ is the *n*-dimensional vector of phenotype data for each ASV, that is, the BLUEs of the ASV within a particular environment (such as KF or YL); *n* is the number of genotypes; ${\mathbf{\upbeta }}$ is the *k*-dimensional vector of fixed covariates, including the common intercept; and ${{X}}$ is the corresponding *n* × *k* design matrix. $a$ represents the additive effect of the marker being tested; ${\mathbf{m}}$ is the *n*-dimensional vector of marker profiles for all genotypes, where each element is coded as 0, 1 or 2, indicating the number of minor alleles at each SNP. ${\mathbf{g}}$ is an *n*-dimensional random vector representing the genetic background effects, with **g** ≈ *N*(0, ${{G}}{\sigma }_{g}^{2}$), where ${\sigma }_{g}^{2}$ is the genetic variance component, and *G* is the VanRaden genomic relationship matrix^56^. ${{e}}$ is the residual term, with *e* ≈ *N*(0, ${{I}}{\sigma }_{e}^{2}$), where ${\sigma }_{e}^{2}$ is the residual variance component and ${{I}}$ is the *n* × *n* identity matrix. After solving the linear mixed model, the marker effect was tested using the Wald test statistic, $W={\hat{a}}^{2}/\mathrm{var}(\hat{a})$, which approximately follows a *χ*^2^ distribution with one degree of freedom. To reduce computational load, the ‘population parameters previously determined’ method^57^ was used, where the variance parameters (${\sigma }_{g}^{2}$ and ${\sigma }_{e}^{2}$) were estimated once, and these fixed values were used throughout the testing procedure for efficient calculation of the test statistics for each marker. All analysis procedures were implemented using R codes developed by ourselves. The variance parameters were estimated by a Bayesian method using the BGLR package (v1.1.1)^58^.

For each bacterial ASV, significant marker–trait associations were identified using a threshold of *P* < 0.05 after FDR (false discovery rate) correction for multiple testing^59^. The corrected *P* value was used to generate the Manhattan plot, while the original *P* value was used for quantile–quantile (QQ) plot.

Considering the linkage disequilibrium between significant SNPs and their neighbouring SNPs, the significant SNPs were grouped into independent QTL regions. The following steps were performed to identify the QTL intervals: First, the genomic positions of upstream and downstream linkage disequilibrium boundaries for each significant SNP were determined based on the linkage disequilibrium decay observed between the significant SNPs and their neighbouring SNPs. Specifically, the boundary was defined as the genomic position where the linkage disequilibrium first dropped below a threshold of 0.1. Then, each significant SNP was assigned its own QTL boundary, based on the identified linkage disequilibrium boundaries. Next, candidate QTL regions that overlapped or had a distance of less than 50 kb between peak SNPs (which corresponds to the average distance where linkage disequilibrium decays to 0.1) were merged into independent QTL regions. These merged QTL regions were represented by the most significant SNP within each region, referred to as the ‘lead SNP’.

### Identification of local eQTL and distant eQTL hotspots

To detect associations between SNPs and gene expression, a linear mixed model assumes that the residuals of expression values follow a normal distribution within each genotype class. However, this assumption can be violated by outliers or non-normality in gene expression data derived from sequencing reads. To address this, gene expression values were normalized using the QQ-normal method from the R package. For both the KF and YL environments, a dataset comprising 17,006 genes was used, with gene expression levels treated as expression traits for eGWAS analyses. After imputing the RNA-seq SNP data, a total of 239,172 and 292,839 RNA-seq SNPs with a MAF ≥ 0.05 and a genotype heterozygosity rate of ≤95% were used for eGWAS in KF and YL, respectively. The SNP-gene expression association was analysed using a standard *Q* + *K* mixed linear model^55^, as described earlier, with a VanRaden genomic kinship matrix^56^ to control for population stratification. For each gene expression traits, significant associations between SNPs (termed eSNPs) and gene expression levels (termed eGenes) used a significance threshold of *P* < 0.05 after Bonferroni–Holm correction^60^. More precisely, the number of tests was equal to the number of SNPs (239,172 for KF and 292,839 for YL), resulting in thresholds of 2.09 × 10^−7^ for KF and 1.71 × 10^−7^ for YL, respectively. Finally, to identify the genomic regions that significantly affect gene expression, we traced each significant eSNP to its associated eQTL, following the same approach described for QTL analysis above.

To classify eQTL as local or distant, the physical genomic regions of the eQTL and their target genes were compared. If the start of the eQTL was within 50 kb downstream of the eGene, or the end of eQTL was within 50 kb upstream of the eGene, it was classified as a local eQTL. Otherwise, it was considered a distant eQTL. To identify potential distant eQTL hotspot regions in KF and YL, the hot_scan method^61^ was applied to all eQTL genomic regions for each chromosome, using the following parameters: -m 100000 -s 0.01. Distant eQTL hotspots were visualized using the circlize package (v0.4.13) in R^62^.

### Transcriptome‑wide association study for ASVs

TWAS integrate GWAS with gene expression datasets to identify gene–trait associations^63^. In this study, we tested the association between gene expression and rhizosphere bacterial microbiomes by treating the abundance of bacterial ASVs as a quantitative trait and the expression levels of 17,006 genes at KF and YL as explanatory variants. The association analysis was performed using a standard *Q* + *K* mixed linear model^55^. To control for hidden batch effects or other confounders in the gene expression data and account for the structure of multiple relatedness levels, we used a gene expression-derived kinship matrix, calculated as ${K}_{\exp }=M{M}^{{\prime} }/c$, where $M$ is the matrix of standardized gene expression features and $c$ is the mean of all diagonal elements in $M{M}^{{\prime} }$.

Following the same procedure to solve the mixed linear model, *P* values were calculated for all genes for each ASV. These raw *P* values were adjusted per FDR method using the p.adjust() function in R v4.1.0 (refs. ^59,64^). Significant associations were identified with an FDR threshold of 5%. For GWAS, eGWAS and TWAS, QQ plots confirmed proper genomic background control in the mixed linear model, ensuring no inflation in false-positive associations.

### Construction of the ASV–GWAS–eGWAS–TWAS regulated network

We constructed a genomic regulating ASV network based on the identified QTL, hotspot regions and significant TWAS genes. The connections between nodes, including QTLs, hotspots, genes and ASVs were visualized using Gephi v0.10 (https://github.com/gephi/gephi). In addition, we developed the Bn-ASV-QTL-eQTL-Gene database (Supplementary Datasets 7–10) to integrate and organize the network data.

### Prediction for ASVs and ionomic traits using multiple omics data

To explore the potential of predicting ionomic traits in rapeseed, data from three omics layers—genomic, transcriptomic and microbiomics—were used for prediction in two distinct locations (KF and YL). These datasets were collected from 175 *B. napus* accessions, each of which was genotyped using WGS^44^. RNA-seq was subsequently performed for the same accessions, with 150 bp pair-end Illumina reads generated for each location. From these sequencing efforts, 752,414 SNPs were obtained from WGS, 243,209 SNPs from RNA-seq in KF, and 295,925 SNPs from RNA-seq in YL. Moreover, 52,962 gene expression features (transcriptomic data) were collected. For microbiome analysis, bacterial ASVs were profiled in the rhizosphere of the rapeseeds, with 2,319 highly abundant ASVs detected at each location.

After filtering out non-polymorphic SNPs and applying Beagle v5.2^54^ imputation, we obtained 637,823 SNPs from WGS, 241,558 SNPs from RNA-seq in KF and 295,925 SNPs from RNA-seq in YL. In addition, 17,006 genes and 203 highly heritable ASVs were selected for subsequent prediction analysis at both KF and YL. We aimed to evaluate the prediction ability for 13 ionome traits (Al, B, Ca, Cu, Fe, K, Mg, Mn, Na, P, Zn, N and C).

For each location, ionome phenotypes were predicted using combinations of the following omics data: genomic data (WGS SNPs), transcriptomic data (RNA SNPs and gene expressions) and bacterial microbiome data (ASVs). This resulted in 15 distinct prediction scenarios, which were classified based on the number and type of omics data combined: single omics data—WGS SNPs (WGS), RNA SNPs (RNA), gene expression (Gene) and microbiome ASVs (ASV); two-omics data combinations—WGS + RNA, WGS + Gene, WGS + ASV, RNA + Gene, RNA + ASV and Gene + ASV; three-omics data combinations—WGS + RNA + Gene, WGS + RNA + ASV, WGS + Gene + ASV and RNA + Gene + ASV; four-omics data combination—WGS + RNA + Gene + ASV. Each model was evaluated for its ability to predict ionome traits within the specific environmental contexts of KF and YL. The following model can be described uniformly within each location:

$${\mathbf{y}}={1}_{n}\mu +\mathop{\sum }\limits_{i=1}^{k}{\mathbf{m}}_{i}+e,$$

where ${\mathbf{y}}$ is an *n*-dimensional vector representing the ionome phenotypic records (that is, BLUEs within a specific location, where *n* is the number of genotypes). ${{\mathbf{m}}}_{i}$ denotes an *n*-dimensional vector of trait values for all individuals, determined by a combination of omics data in a given case. The index *k* represents the number of omics data combinations; that is, 1 denotes single omics data, and 2 or 3 or 4 denotes two or three or four omics data combinations. *e* represents the residual term, assumed to follow a normal distribution *e* ≈ *N*$(0,{{I}}{\sigma }_{e}^{2})$, with ${\sigma }_{e}^{2}$ as the residual variance component, and *I* is the *n* × *n* identity matrix. We assume that **m**_*i*_ ≈ *N*$(0,{{{V}}}_{i}{\sigma }_{{\mathbf{m}}_{i}}^{2})$, where ${\sigma }_{{\mathbf{m}}_{i}}^{2}$ is the corresponding variance component, and ${{{V}}}_{i}$ is the VanRaden genomic relationship matrix^56^ for WGS and RNA SNPs data. For gene expression and ASV data, ${{{V}}}_{i}$ is a covariance matrix derived from the corresponding omics data. Assuming that ${{{M}}}_{i}$ is the *n* × *t* matrix of standardized features of gene expression or ASVs (*t* is the number of features), we have ${V}_{i}={M}_{i}{{M}_{i}}^{{\prime} }/{c}_{i}$ where ${c}_{i}$ is the mean of all diagonal elements in the matrix ${M}_{i}{{M}_{i}}^{{\prime} }$.

The prediction ability was assessed using a fivefold cross-validation approach. Prediction models were implemented through the R package BGLR^58^. The cross-validation process was repeated 100 times, and the prediction accuracy was evaluated using the Pearson correlation coefficient between the observed and predicted ionome phenotypic values. Similarly, a comparable strategy was used to predict the abundance of ASVs within each location. Using three types of omics data—WGS SNPs (WGS), RNA SNPs (RNA) and gene expression data (Gene)—we created seven combinations of input data for predicting the ASVs: WGS, RNA, Gene, WGS_RNA, WGS_Gene, RNA_Gene and WGS_RNA_Gene. For ASV prediction, the same fivefold cross-validation framework was applied, and the prediction ability was evaluated using the Pearson correlation coefficient between observed and predicted ASV abundances.

### Transcriptome, bacterial microbiome and ionome correlation analysis

To estimate the correlation between root gene expression, ionome accumulation and rhizosphere bacterial microbiome assembly, we performed a Mantel statistical test on the data matrices. Euclidean distance was calculated for both root gene expression and ionome accumulation, while Bray–Curtis distance was used to quantify bacterial composition. The Pearson correlation method was applied through the ‘mantel’ function in the vegan package (v2.6.4) of R. A total of 1,999 permutations were performed to evaluate the statistical significance of the correlations.

### Functional characterization of genes associated with *Sphingopyxis*

To investigate the function of genes associated with the genus *Sphingopyxis*, we performed GO enrichment analyses with the genes identified through GWAS and TWAS analyses. GO terms were enriched using Goatools (v1.3.1) (https://github.com/tanghaibao/goatools), with terms showing significant enrichment (FDR < 0.05) compared to the whole-transcriptome background.

### Whole genome assembly and annotation for *Sphingopyxis*

To enhance our understanding of the genetic and genomic information of *Sphingopyxis*, we performed a de novo whole-genome assembly of the bacterial *Sphingopyxis* isolate 31, using both Nanopore and Illumina platforms. Bioinformatic analyses were performed through the online Majorbio Cloud (http://cloud.majorbio.com)^65^, provided by Shanghai Majorbio Bio-pharm Technology. The detailed procedures are as follows. The raw paired-end Illumina sequencing reads were quality filtered using fastp (v0.23.0). Nanopore reads were extracted, basecalled and demultiplexed, and trimmed using ONT Guppy, with a minimum *Q*-score cut-off of 7. Then the high-quality clean reads were co-assembled to construct complete genomes using Unicycle (v0.4.8)^66^. The final assembly was polished using Pilon (v1.22) with short-read alignments to reduce the rate of small errors. The coding sequences of both the chromosome and plasmid were predicted separately using Prodigal (v2.6.3)^67^. Transfer RNAs were predicted using tRNA-scan-SE (v 2.0)^68^, and rRNAs were identified using Barrnap (v0.9) (https://github.com/tseemann/barrnap). The predicted coding sequences were annotated using the COG (https://www.ncbi.nlm.nih.gov/research/COG) and Kyoto Encyclopedia of Genes and Genomes (KEGG) (https://www.genome.jp/kegg/kegg1.html) databases. Sequence alignment tools such as Diamond (v2.0.15)^69^ and HMMER (v3.3.1)^70^ were used for alignment, and the best-matched subjects (*e* < 10^−5^) were selected for gene annotation.

### High-throughput bacterial cultivation and identification by Illumina sequencing

To obtain representative bacterial strains for functional validation, we performed high-throughput bacterial cultivation^36^ using two rapeseed varieties, YL29 and YL260, which were grown in natural soil at the experimental station at YL, where one of the field experiments took place. These two genotypes were collected at the flowering stage to align with our field sampling protocol. In detail, roots from three representative plants of each variety, each with bacteria colonized, were freshly dug up and thoroughly mixed to minimize variation between samples. The roots were washed with sterilized water and further rinsed three times in sterile phosphate-buffered saline (PBS) buffers on a shaker at 180 r.p.m. for 15 min. Then the root tissue was ground into a homogeneous slurry, which was then transferred into MgCl_2_ and allowed to sediment for 15 min. The supernatant was diluted, distributed and cultivated in 96-well cell culture plates containing a 1:10 (*v*/*v*) dilution of tryptic soy broth (TSB). The cultures were incubated for 15 days at room temperature. Preliminary experiments determined the approximate dilution gradients, and a 6,000× dilution was used for subsequent bacterial identification for both genotypes, YL29 and YL260.

To identify pure bacterial cultures, we performed a two-sided barcoded PCR followed by sequencing of the bacterial 16S rRNA (V5–V7) gene using Illumina MiSeq. DNA was extracted from the cultivated bacteria using the alkaline lysis method^36^. Specifically, 6 μl of bacterial cultures was added to 10 μl of lysis buffer I, containing 25 mM NaOH and 0.2 mM Na_2_–EDTA at pH 12, and incubated at 95 °C for 30 min. The pH value was then adjusted to 7.5 by adding 10 μl of buffer II, containing 40 mM Tris–HCl. Each position on the 96-well plates was indexed using a two-step PCR protocol with degenerate primers 799F and 1193R, each containing well- and plate-specific barcodes to amplify the variable regions V5–V7. In the first PCR step, the primers were used to amplify the bacterial 16S rRNA gene. For quality control, the negative control (nuclease-free water) and positive control (*Escherichia coli* DNA) were added to wells A12 and B12, respectively. The PCR reactions for each well were conducted according to the established protocol. The first PCR products were diluted 40× and used as templates in a second PCR, where the products were labelled with barcoded primers. The PCR reaction system and cycling conditions followed the previously described methods^36^. The second PCR products were purified using the Wizard SV Gel and PCR Clean-up System (Promega) and the Agencourt AMPure XP Kit (Beckman Coulter). DNA concentrations were measured using the Quant-iT PicoGreen dsDNA Assay Kit (Life Technologies), and the samples were pooled in equal amounts. A total of 1,500 ng of purified PCR product libraries were sequenced on an Illumina MiSeq platform. Each sequence contained a plate barcode, a well barcode and the V5–V7 regions of the 16S rRNA gene. Bioinformatic analysis to identify the bacterial taxa was performed using an established pipeline^36^.

### Purification and preservation of cultivated bacteria

To identify specific ASVs from the cultivated bacteria, five wells containing the corresponding bacterial cultures were selected and inoculated onto solid media for three consecutive rounds of purification. After this, an individual colony was picked and used for liquid cultures. These liquid cultures were then subjected to Sanger sequencing of the bacterial 16S rRNA gene sequence by using the primers 27F (AGAGTTTGATCCTGGCTCAG) and 1492R (TACGGCTACCTTGTTACGACTT). In addition to sequencing, the bacterial cultures were preserved by preparing glycerol stocks. Phylogenetic relationships of the cultivated bacterial isolates were determined using MEGA11 software (version 11.0).

### Metabolome analysis of root system following *Sphingopyxis* inoculation

To investigate the potential impact of *Sphingopyxis* inoculation on the metabolic feature of the root system, we used an untargeted metabolomics approach on rapeseed plants grown in the same natural soil as described above. In detail, the rapeseeds were cultivated under two nitrogen conditions: half nitrogen (2.375 mM KNO_3_, 11.775 mM KCl, 313 μM KH_2_PO_4_, 750 μM CaCl_2_, 50 μM H_3_BO_3_, 50 nM CuSO_4_·5H_2_O, 50 μM MnSO_4_·H_2_O, 2.5 μM KI, 375 μM MgSO_4_, 52.5 nM CoCI_2_·6H_2_O, 44 μM FeNaEDTA, 0.5 μM Na_2_MoO_4_·2H_2_O, 15 μM ZnSO_4_·7H_2_O) and full nitrogen (4.75 mM KNO_3_, 9.4 mM KCl, 313 μM KH_2_PO_4_, 750 μM CaCl_2_, 50 μM H_3_BO_3_, 50 nM CuSO_4_·5H_2_O, 50 μM MnSO_4_·H_2_O, 2.5 μM KI, 375 μM MgSO_4_, 52.5 nM CoCI_2_·6H_2_O, 44 μM FeNaEDTA, 0.5 μM Na_2_MoO_4_·2H_2_O, 15 μM ZnSO_4_·7H_2_O) for genotypes YL29 and YL260 over a 28 day period. Half of the plants were inoculated with a *Sphingopyxis* isolate obtained from the earlier cultivation. Before inoculation, we confirmed the identity of the *Sphingopyxis* isolate by comparing their 16S rRNA sequence with the ASVs generated from the field experiments using HISAT2 (ref. ^49^). The preparation and inoculation followed the methods outlined in our previous work^32^. For sample collection, soil was thoroughly washed off the entire root system, and the roots were rinsed three times with sterile water. The lateral roots were then excised, dried with clean tissue and immediately frozen in liquid nitrogen. Approximately 100 mg of lateral roots was ground into a fine powder in liquid nitrogen and mixed with prechilled 80% methanol. The mixture was vortexed and incubated on ice for 5 min. Afterwards, the sample was centrifuged at 15,000 *g*, 4 °C for 20 min. The supernatant was diluted with LC–MS grade water to a final concentration of 53% methanol, transferred to a fresh Eppendorf tube and centrifuged again at 15,000 *g*, 4 °C for 20 min. Finally, the supernatant was injected into the LC–MS/MS system for analysis^71^.

The samples were analysed using an ExionLC AD system (SCIEX) coupled with a QTRAP 6500+ mass spectrometer (SCIEX) at Novogene. A 20 min linear gradient was applied to inject samples onto an Xselect HSS T3 (2.1 × 150 mm, 2.5 μm) at a flow rate of 0.4 ml min^−1^ operating in both positive and negative polarity modes. The mobile phases were the following: eluent A (0.1% formic acid in water) and eluent B (0.1% formic acid in acetonitrile)^72^. The gradient elution protocol was as follows: 2% B, 2 min; 2–100% B, 15 min; 100% B, 17 min; 100–2% B, 17.1 min; 2% B, 20 min. The QTRAP 6500+ mass spectrometer was operated in both negative and positive ionization modes with the following settings: curtain gas at 35 psi, collision gas set to medium, ion source temperature at 550 °C, ion source gas of 1:60, ion source gas of 2:60, and ionspray voltage at 5,500 V in positive mode and −4500 V in negative mode. Targeted metabolite analysis using multiple reaction monitoring was applied to quantify IAA and its precursors in *Sphingopyxis* culture supernatants. Separately, untargeted LC–MS profiling in full-scan mode was conducted on root tissues to detect broader metabolic shifts associated with microbial inoculation. For quantitative analysis, multiple reaction monitoring transitions were monitored based on the Q3 (product ion) signal. For qualitative confirmation of metabolite identity, Q1 (precursor ion), Q3, retention time, declustering potential and collision energy (35 eV in positive mode and −30 eV in negative mode) were matched against the in-house reference database. The HPLC–MS/MS data files were processed with SCIEX OS version 1.4 and peak integration and correction was performed under the following parameters: minimum peak height = 500, signal-to-noise ratio = 5, and Gaussian smooth width = 1. The area of each peak was used to represent the relative content of the corresponding metabolite. Annotation of metabolites was based on the KEGG database (http://www.genome.jp/kegg/). Partial least squares discriminant analysis was conducted using metaX (v2.3.0)^73^. Statistical significance was estimated using univariate analysis (Student’s *t*-test). Differential metabolites were defined as having variable importance in projection > 1, *P* < 0.05 and fold change ≥2 or ≤0.5. Metabolic pathway enrichment was considered significant when the ratio satisfied *x*/*n* > *y*/*N*, with *P* < 0.05 for the metabolic pathway. Here, *x* denotes the number of significantly altered metabolites mapped to a given pathway, *n* the total number of significantly altered metabolites, *y* the total number of metabolites assigned to that pathway in the reference database and *N* the total number of background metabolites used for enrichment analysis.

### Confocal microscopy for DR5::VENUS expression in the root

*A. thaliana* Columbia-0 (Col-0) was used in this study. To prepare the seeds, they were surface sterilized by soaking in 75% (*v*/*v*) ethanol for 2 min, followed by three washes with sterile water. Seeds were then immersed in 10% (*v*/*v*) sodium hypochlorite for 5 min and washed five times with sterile water. Individual bacterial strains were cultured in TSB medium (Oxoid) for 24 h. Individual bacterial strains were centrifuged, washed twice, resuspended in sterile water and adjusted to a final density of 10^8^ c.f.u. ml^−1^ (optical density at 600 nm (OD_600_) = 0.5). The sterilized seeds were then sown on plates (13 cm × 13 cm) containing 50 ml of 1/4 Murashige and Skoog medium (Coolaber), supplemented with 10 g l^−1^ sucrose and 10 g l^−1^ agar. Following a 2 day stratification period, plates were placed vertically in a growth chamber under a long-day photoperiod (16 h:8 h, light/dark, relative humidity 60%) at 22 °C. The roots of 2-day-old seedlings were inoculated with 8 μl of the bacterial inoculum. GFP staining was carried out 3 h and 3 days after inoculation. The GFP signal was observed under a light microscope (Leica) according to the manufacturer’s instructions.

### Scanning electron microscopy for bacterial colonization

Surface-sterilized rapeseed seeds were germinated on germ-free plates. Ten-day-old seedlings were then transferred to sterile plastic bottles containing 50 ml of 1/4 Murashige and Skoog medium, with sterile foam rods placed inside the bottles to support the seedlings. At the same time, 1 ml of bacterial suspension (OD_600_ = 0.5) was inoculated into the experimental group, while the control received an equal volume of sterile water. After 3 days of co-cultivation, the seedlings were removed, washed three times with PBS and fixed in 2.5% sterile glutaraldehyde at 4 °C for 12 h. The samples were then dehydrated in a graded series of ethanol (30%, 50%, 70%, 85% and 90%) for 15 min each, followed by two 15 min washes in anhydrous ethanol. After dehydration, the samples were freeze dried. Finally, the bacterial colonization on the root surfaces was observed using a scanning electron microscope (JSM-5610, Phenom) at 10 kV.

### Nitrogen phenotype screening in the agar–plate system

The seeds of genotypes YL29 and YL260 were surface sterilized by soaking in 75% (*v*/*v*) ethanol for 2 min, followed by three washes with sterile water. The seeds were then soaked in 10% (*v*/*v*) sodium hypochlorite for 5 min and washed five times with sterile water. Individual bacterial strains were cultured in TSB medium (Oxoid) for 24 h, after which the bacterial cells were centrifuged, washed twice, and resuspended in sterile water to a final concentration of 10^8^ c.f.u. ml^−1^ (OD_600_ = 0.5). The sterilized seeds were sown on plates (25 cm × 25 cm) containing 200 ml of 1/4 Murashige and Skoog medium (Coolaber), supplemented with 10 g l^−1^ sucrose and 10 g l^−1^ agar. The nitrogen concentration in the medium was adjusted to 100% nitrogen and 50% nitrogen. In brief, the Murashige and Skoog medium was autoclaved at 120 °C for 20 min, and then, when the temperature dropped to approximately 55 °C, 1 ml of bacterial suspension or 1 ml of sterile water (as mock control) was added. The mixture was shaken thoroughly and poured into the plates. The seeds were evenly distributed on the plates, which were sealed with Parafilm and incubated in a growth chamber under a long-day photoperiod (16 h:8 h, light/dark, relative humidity 60%) at 25 °C. After 9 days of growth, the root traits including the total length, lateral root density and shoot traits including biomass and nitrogen accumulation were investigated according to Yu et al.^32^.

### Auxin detection and measurement using different substrates by HPLC–MS/MS

*Sphingopyxis* isolate 31 was inoculated into half-strength TSB liquid medium, with or without 100 mg l^−1^ Trp, IAN, IAM and TAM and cultured at 28 °C for 48 h. The bacterial culture was centrifuged at 6,000 *g* for 6 min to obtain the bacterial supernatant, which was then filtered through a 0.22 μm pore diameter filter (JinTeng) to completely remove the bacteria. The content of IAA in the bacterial supernatant was measured by Ruiyuan Biotechnology. In detail, samples were thawed and sonicated for 10 min, and a 5 ml aliquot was spiked with 80 µl of deuterated IAA internal standard before extracting twice with dichloromethane at 4 °C to separate the organic phase, which was evaporated under nitrogen and reconstituted with methanol and water (200 µl each); the solution was centrifuged, filtered through a 0.22 µm membrane and stored at −20 °C. The extracts were analysed using a QSight LX 50 UPLC coupled to a QSight 420 triple-quadrupole mass spectrometer equipped with a Poroshell 120 EC-C18 column (2.1 × 100 mm, 2.7 µm) and a matching guard column, maintained at 40 °C with a flow rate of 0.3 ml min^−1^ and 10 µl injection volume; gradient elution with water containing 0.02% formic acid and methanol separated hormones over 15 min. Detection used electrospray ionization and multiple-reaction monitoring (positive/negative switching), with drying gas at 120 ml min^−1^, nebulizer gas at 240 ml min^−1^, interface temperature of 300 °C and spray voltage of +5,500 V or −5,000 V, while specific parent–product ion transitions were optimized for each analyte to ensure high-sensitivity quantification. Data acquisition relied on monitoring at least two fragment ions per hormone and comparing retention times and response ratios with standards; concentrations were calculated from calibration curves constructed by plotting the ratio of analyte peak area to internal-standard peak area against standard concentrations. Calibration solutions were prepared from a 1 mg ml^−1^ stock by diluting to 0.1–200 ng ml^−1^, each containing 20 ng ml^−1^ internal standard, and raw data along with the standard curves and supporting figures are provided in the attached files.

### Nitrogen fixation capacity of bacterial *Sphingopyxis* isolate

To test the nitrogen fixation capacity of *Sphingopyxis* isolate 31, a 5 μl suspension (OD_600_ = 0.50) of the bacterial culture (sterile water was used as a mock control) was spread onto a nitrogen-free agar plate (Ashby’s medium) that contained KH_2_PO_4_ (0.2 g), MgSO_4_ (0.2 g), NaCl (0.2 g), CaCO_3_ (5.0 g), mannitol (10.0 g), CaSO_4_ (0.1 g) and agar (15 g) dissolved in 1,000 ml distilled water at pH 7.0 ± 0.1. The inoculated plates were incubated at 28 °C for 72 h, and colony growth was then observed by root system scanner (Epson perfection V850 Pro).

### Functional validation experiments in soil pots

For the pot experiment, the soil matrix (soil/vermiculite/perlite = 6:3:1) was sterilized twice, with a 24 h interval. The seeds of genotypes YL29 and YL260 were surface sterilized according to the method described above and cultivated on the plate for 24 h until germination. Bacterial cells were then centrifuged, washed twice and resuspended in phosphate buffer, adjusting the final density to 10^8^ c.f.u. ml^−1^ (OD_600_ = 0.5). The germinated seeds were immersed in the bacterial suspension for 2 h, while the control seeds were immersed in sterile phosphate buffer. After that, four germinated seedlings were planted in each pot containing 200 g of soil in a growth chamber under a long-day photoperiod (16 h:8 h, light/dark, relative humidity 60%) at 25 °C. After transplantation, each seedling was watered with 2 ml of the bacterial suspension, while the control seedlings were watered with 2 ml of phosphate buffer. After 15 days, 5 ml of nutrient solution with varying nitrogen gradients was poured into each pot, and the soil was kept moist by daily watering. The root and shoot samples were collected and processed after 28 days of cultivation as previously described.

### Verification of bacteria–bacteria interactions using SynCom

A total of 12 bacterial strains, including isolate 31, were used to construct the synthetic bacterial community (SynCom). Each strain was streaked on TSB agar medium, and single colonies were selected for liquid culture. The bacterial cultures grew to an optical density (OD_600_) of 0.5 for subsequent use. For the SynCom, equal volumes of the cultures were mixed to prepare a 2 ml bacterial suspension. The soil matrix (soil/vermiculite/perlite = 6:3:1) was sterilized twice with a 24 h interval. The seeds of rapeseed genotypes YL29 and YL260 were surface sterilized as described earlier and placed on plates to germinate for 24 h. After germination, the seedlings were immersed in the SynCom bacterial suspension for 2 h, while control seedlings were immersed in sterile phosphate buffer. Germinated seedlings were then planted in pots containing 200 g of sterilized soil in a growth chamber under a long-day photoperiod (16 h light/8 h dark, 60% relative humidity, 25 °C). On days 10 and 20 after sowing, each seedling was watered with 2 ml of the SynCom bacterial suspension, while the control plants received 2 ml of phosphate buffer. Following a 15 day incubation, 5 ml of nutrient solution with different nitrogen gradients was applied to each pot, and daily watering kept the soil moist. After 28 days, root and shoot samples were collected for further analysis. The shoots of rapeseed plants were placed in envelopes and heated at 105 °C for 30 min to deactivate chlorophyll, followed by drying at 65 °C until a constant weight was reached. The dried plant material was ground into a fine powder for subsequent analysis. A 0.2 g sample of the dried material was placed in a digestion tube, and 7 ml of digestion solution (5 ml H_2_SO_4_ and 2 ml H_2_O_2_) was added. Nitrogen content in the plant material was determined using a Kjeldahl nitrogen determinator.

### Validation of gene–bacterial interactions using *Arabidopsis* mutants

*A. thaliana* accession Col-0 serves as the wild-type genotype. The *Arabidopsis* transfer DNA (T-DNA) insertion mutants used were as follows: AT1G60940 (SALK_056781C), At4g37800 (SALK_201184C) and At1g64620 (SALK_202973C). *Arabidopsis* seeds were surface sterilized by immersing them in 75% (*v*/*v*) ethanol for 2 min, followed by three washes with sterile water. The seeds were then soaked in 10% (*v*/*v*) sodium hypochlorite for 5 min, followed by five additional washes with sterile water. Sterilized seeds were sown on plates containing 50 ml of 1/4 Murashige and Skoog medium supplemented with 10 g l^−1^ sucrose and 10 g l^−1^ agar, with or without *Sphingopyxis* isolate 31. In the medium, we defined the nitrogen concentrations as 100% nitrogen and 50% nitrogen, where 50% nitrogen corresponds to half the concentration of 100% nitrogen. After 2 days of stratification, the plates were placed vertically in a growth chamber under a long-day photoperiod (16 h light/8 h dark, relative humidity 60%) at 22 °C. Biomass and nutrient determinations were performed according to previously defined protocols^33^.

### Real-time PCR quantification of gene expression

Total RNA was extracted using TRIzol reagent (TAKARA) according to the manufacturer’s instructions. A total of 1,000 ng of RNA was used for reverse transcription with the Transcriptor First Strand cDNA Synthesis Kit (TAKARA). The resulting cDNA was diluted 10 times and used as a template for real-time quantitative PCR. The expression levels of BnaA01g23120D and BnaC07g46780D were measured using the following primers: BnaA01g23120D (forward, TCGATGAGAACGTGGCAAGG; reverse, GCTCAAATAGCTCGCCTCCA) and BnaC07g46780D (forward, AGTGCGTACCACCAATTGACT; reverse, GGGAGGGACAGGAAAACGAG). Quantitative PCR was performed on a Thermo Fisher 7500 RT-PCR system using the following reaction mix: 0.2 μl of forward primer (10 μM), 5 μl of SYBR Premix Ex Taq (2×), 1 μl of cDNA template, 0.2 μl of reverse primer (10 μM) and distilled and deionized water to a final volume of 10 μl. BnaActin2 (forward, AGAGGTTCTGTTCCAGCCGT; reverse, TGCTCATACGGTCCGCAATA) was used as the reference gene. Relative gene expression was calculated using the 2^−ΔΔCt^ method.

### Reporting summary

Further information on research design is available in the Nature Portfolio Reporting Summary linked to this article.

## Supplementary information

Supplementary Information Supplementary Figs. 1–16 and Table 1.

Reporting Summary

Supplementary Data 1 Supplementary Datasets 1–14.

## Source data

Source Data Fig. 1 Statistical source data.

Source Data Fig. 2 Statistical source data.

Source Data Fig. 3 Statistical source data.

Source Data Fig. 4 Statistical source data.

## Data Availability

All raw rapeseed RNA-seq and bacterial 16S gene data generated in this study have been deposited in the Sequence Read Archive (http://www.ncbi.nlm.nih.gov/sra) under the BioProject IDs PRJNA986524 (KF, RNA-seq), PRJNA990484 (YL, RNA-sRaeq), PRJNA956663 (KF, 16S) and PRJNA960662 (YL, 16S). Raw format mzML and processed metabolomics data—including peak tables, compound annotations (MSI level 2) and quantification results from both untargeted and targeted assays—are available via figshare at https://figshare.com/s/caa71efed449b6f8c4e0 (ref. ^74^). All data necessary to reproduce the results are included in the processed datasets, and additional materials can be made available upon reasonable request. Source data are provided with this paper.

## Code availability

We deposited customized scripts in a repository available via GitHub at https://github.com/Ligl0226/proj_rapeseed.
