# Supplementary material for: Large-scale multi-omics unveils host–microbiome interactions driving root development and nitrogen acquisition
Source: Nat Plants. 2026 Feb 3;12(2):319–36. doi: 10.1038/s41477-025-02210-7 (PMC12929062; doi:10.1038/s41477-025-02210-7)
Supplement: Supplementary file 1 — Supplementary Figs. 1–16 and Table 1. [file 41477_2025_2210_MOESM1_ESM.pdf]

nature plants

Supplementary information

https://doi.org/10.1038/s41477-025-02210-7

Large-scale multi-omics unveils host–microbiome interactions driving root development and nitrogen acquisition

In the format provided by the authors and unedited

Large-scale multi-omics unveils host-microbiome interactions driving root development and nitrogen acquisition

Nannan Li[1,#,*], Guoliang Li[2,#], Xiaofang Huang[3,4,5#], Lige Ma[1,#], Danning Wang[6,#], Yu Luo[1,#], Xulv Cao[1], Yantao Zhu[7], Jianxin Mu[7], Ran An[7], Jianhua Zhao[7], Yongfeng Wang[8], Cuiling Yang[8], Hao Chen[8], Ying Xu[9], Lixi Jiang[9], Meng Luo[10], Xiaodan Li[10], Yachen Dong[10], Xinping Chen[1,11], Frank Hochholdinger[12], Yong Jiang[2], Jochen C. Reif[2], Daojie Wang[8,*], Yanfeng Zhang[7,*] Yang Bai[5,*], Peng Yu[3,4,*]

[1] College of Resources and Environment, and Academy of Agricultural Sciences, Southwest University, Chongqing 400715, China

[2] Leibniz Institute of Plant Genetics and Crop Plant Research (IPK), Stadt Seeland, Gatersleben 06466, Germany.

[3] Emmy Noether Group Root Functional Biology, Institute of Crop Science and Resource Conservation (INRES), University of Bonn, Bonn 53113, Germany

[4] Plant Genetics, TUM School of Life Sciences, Technical University of Munich (TUM), Freising 85354, Germany

[5] Peking-Tsinghua Center for Life Sciences, College of Life Sciences, Peking University, Beijing 100871, PR China.

[6] Plant Breeding, TUM School of Life Sciences, Technical University of Munich, Freising 85354, Germany

[7] Hybrid Rapeseed Research Center of Shaanxi Province, Yangling 712100, PR China

[8] College of Agriculture, State Key Laboratory of Crop Stress Adaptation and Improvement, Henan University, Kaifeng 475004, Henan, PR China

[9] Institute of Crop Science, Zhejiang University, Hangzhou 310058, PR China

[10] Shanghai Majorbio Research Institute, Shanghai 201203, PR China

[11] Interdisciplinary Research Center for Agriculture Green Development in Yangtze River Basin, Southwest University, Chongqing 400715, PR China

[12] Crop Functional Genomics, Institute of Crop Science and Resource Conservation (INRES), University of Bonn, Bonn 53113, Germany

[#] These authors equally contributed to this work.

[*] To whom correspondence should be addressed:

Nannan Li: linannan2013@swu.edu.cn

Daojie Wang: wangdj@henu.edu.cn

Yanfeng Zhang: zhangyfcl@163.com

Yang Bai: ybai@pku.edu.cn

Peng Yu: pengyu.yu@tum.de (lead contact)

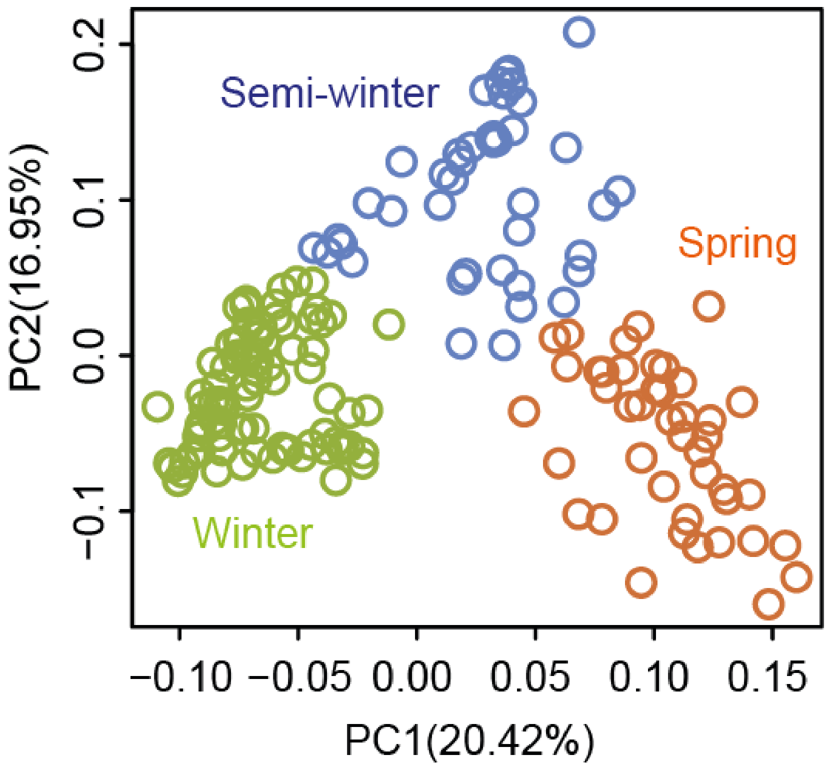

Supplemental Figure 1. Host genetic variation for 300 *B. napus* ecotypes visualized by a principal component analysis using whole genome resequencing data. Colours indicate growth-type classifications: green = Winter, blue = Semi-winter, and orange = Spring ecotypes. PC1 and PC2 explain 20.42% and 16.95% of the genetic variance, respectively. PCA was performed once using filtered SNP markers derived from whole-genome resequencing. Field sampling locations were Kaifeng (North China) and Yangling (Northwest China); n = 175 ecotypes per site were used for downstream analyses after removing site-specific ecotypes. No statistical tests were performed for this visualization.

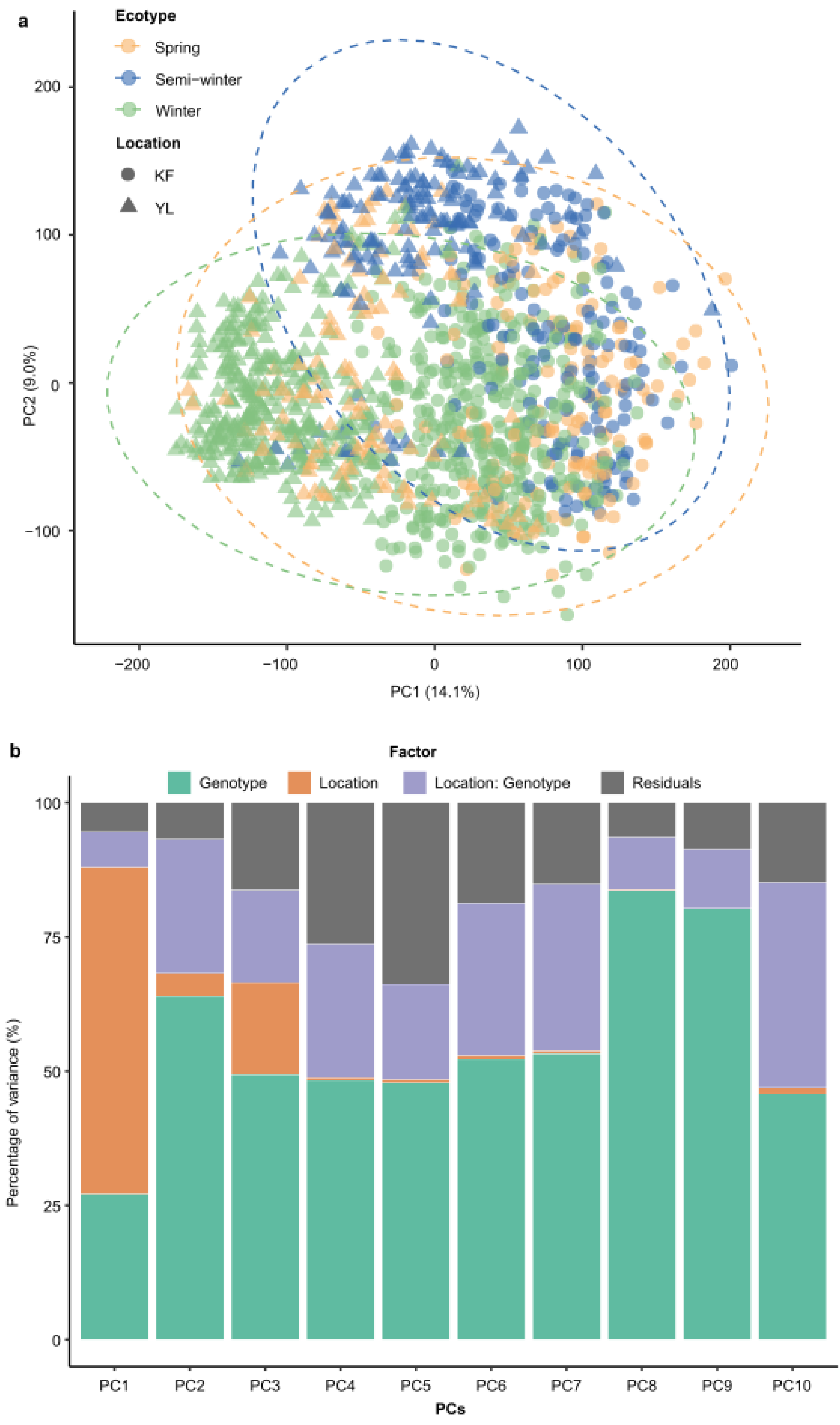
a
Ecotype
Spring
Semi−winter
Winter
Location
KF
YL
PC2 (9.0%)
PC1 (14.1%)
200
100
0
−100
−200
−100
0
100
200
b
Factor
Genotype
Location
Location: Genotype
Residuals
Percentage of variance (%)
100
75
50
25
0
PC1
PC2
PC3
PC4
PC5
PC6
PC7
PC8
PC9
PC10
PCs

Supplemental Figure 2. Overall transcriptome analysis for *B. napus* population (*n* = 175) using all expressed genes. (a) Principal component analysis (PCA) of n = 175 ecotypes based on transcriptome data. Colours indicate ecotype groups (orange = Spring, blue = Semi-winter, green = Winter) and point shapes indicate sampling locations (circles = Kaifeng (KF), triangles = Yangling (YL)). PC1 and PC2 explain 14.1% and 9.0% of the total genomic variance, respectively. (b) Variance partitioning of the first 10 principal components for the same n = 175 ecotypes, showing the relative contributions of Genotype (green), Location (orange), Genotype × Location (purple) and Residuals (grey). Variance components were estimated using a linear mixed model; no statistical tests were performed. Axis labels and colour codes are defined in the figure.

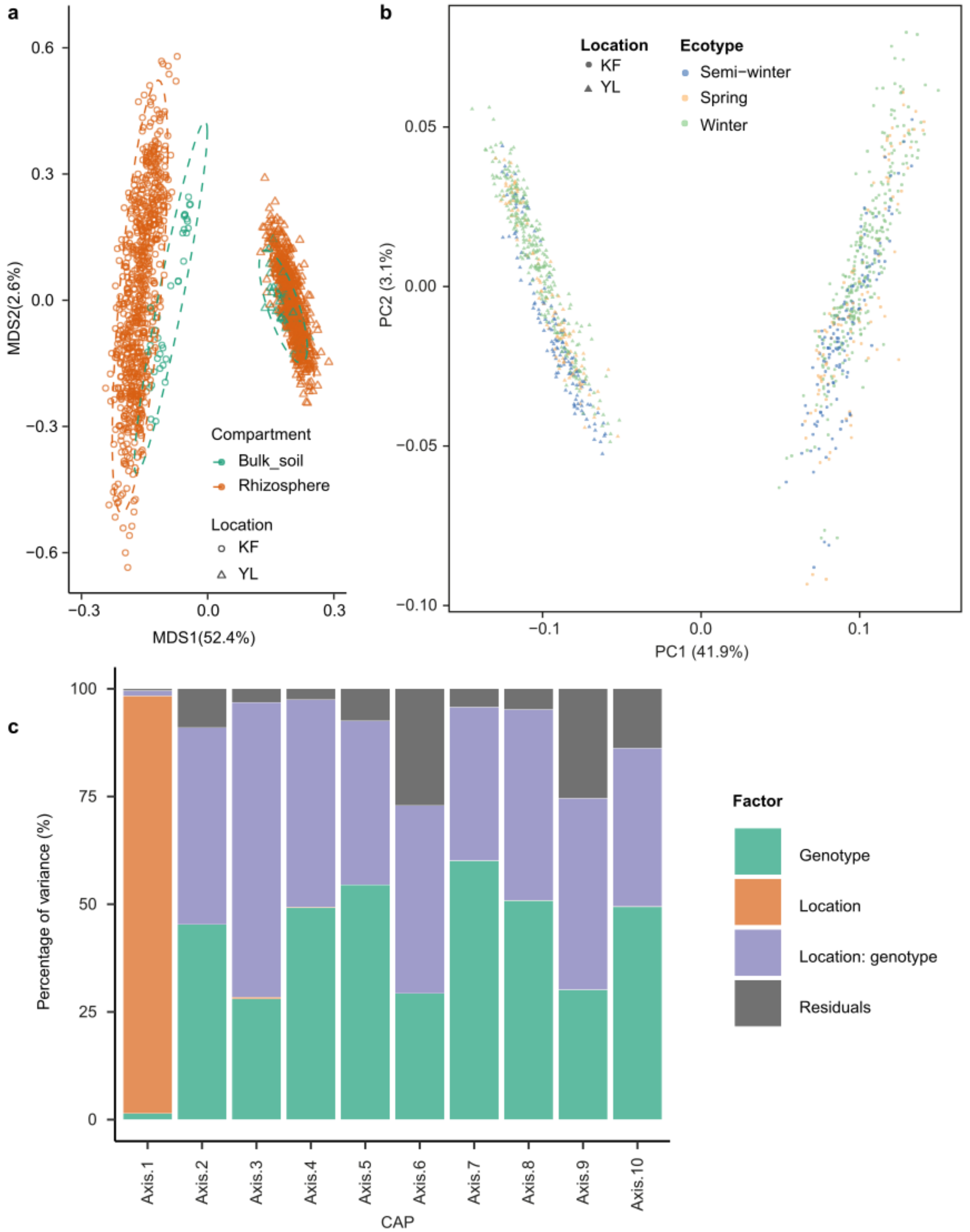

Supplemental Figure 3. Large-scale bacterial microbiome profiling in the B. napus rhizosphere. (a) Multidimensional scaling (MDS) of Bray–Curtis dissimilarities showing the effects of compartment and location on bacterial community composition. Each point represents one sample (Bulk soil, n = 72; Rhizosphere, n = 1341) collected from Kaifeng (KF; circles) and Yangling (YL; triangles). Colours denote sample compartments (green = bulk soil, orange = rhizosphere). MDS1 and MDS2 explain 52.4% and 2.6% of the variance, respectively. No statistical hypothesis tests were applied; the ordination was generated once using highly abundant ASVs as input. (b) Principal component analysis (PCA) of bacterial community profiles across three ecotypes at two locations. Colours represent ecotypes (blue = semi-winter, orange = spring, green = winter) and shapes indicate sampling location (circles = KF, triangles = YL). PC1 and PC2 explain 41.9% and 3.1% of the variance. Each point corresponds to one rhizosphere microbiome sample (n = 1341). PCA was performed once using the same highly abundant

ASVs as input; no statistical tests were applied. (c) Variance partitioning of the first ten CAP axes showing the relative contributions of Genotype (green), Location (orange), Genotype × Location interaction (purple) and Residuals (grey) to variation in bacterial community structure. Variance components were estimated using a linear mixed model; no statistical hypothesis testing was performed.

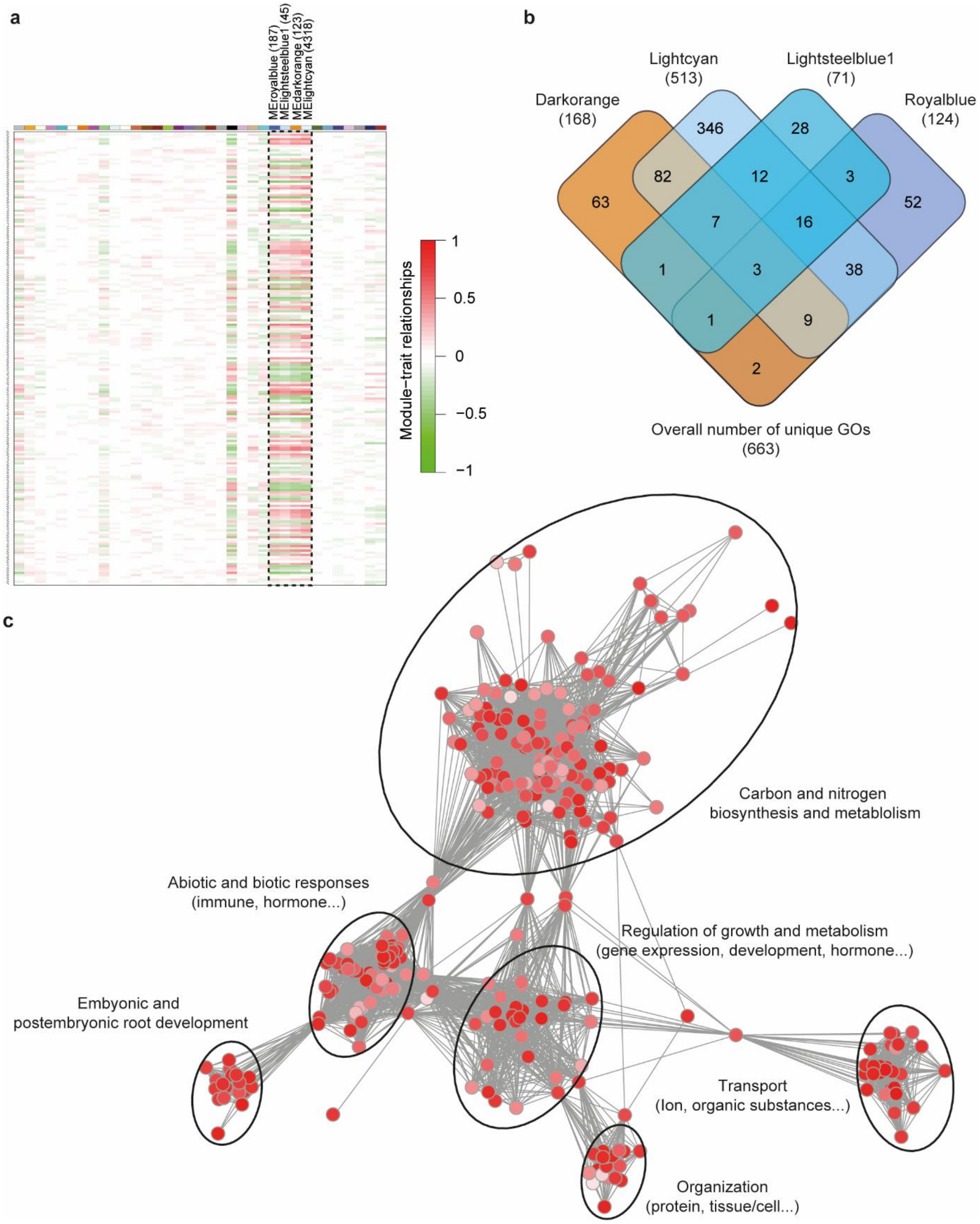

Supplemental Figure 4. Identification of gene co-expressed modules and functional characterization. (a) Weighted gene co-expression network analysis (WGCNA) identified 35 co-expressed gene modules, with the number of genes in each module indicated in brackets. The heatmap displays Pearson correlation coefficients between module eigengenes and the 203 heritable bacterial ASVs (n = 203 ASVs). The colour scale ranges from –1 (green) to +1 (red) and reflects the strength and direction of module–trait relationships. No statistical hypothesis testing was applied beyond correlation estimation. (b) Overlap of enriched Gene Ontology (GO) terms among four highly gene–microbe–

associated modules (Darkorange, Lightcyan, Lightsteelblue1, Royalblue). The number of enriched GO terms per module is indicated in brackets. Numbers within the overlapping regions indicate shared GO terms, while non-overlapping numbers indicate module-specific GO categories. (c) Network visualization of the GO terms shared among the highly correlated WGCNA modules. Nodes represent enriched GO categories and edges connect semantically related terms. Major functional clusters—including carbon and nitrogen metabolism, abiotic and biotic responses, regulation of growth and metabolism, transport, organization, and embryonic/postembryonic root development—were identified based on semantic similarity clustering using REVIGO (http://revigo.irb.hr/).

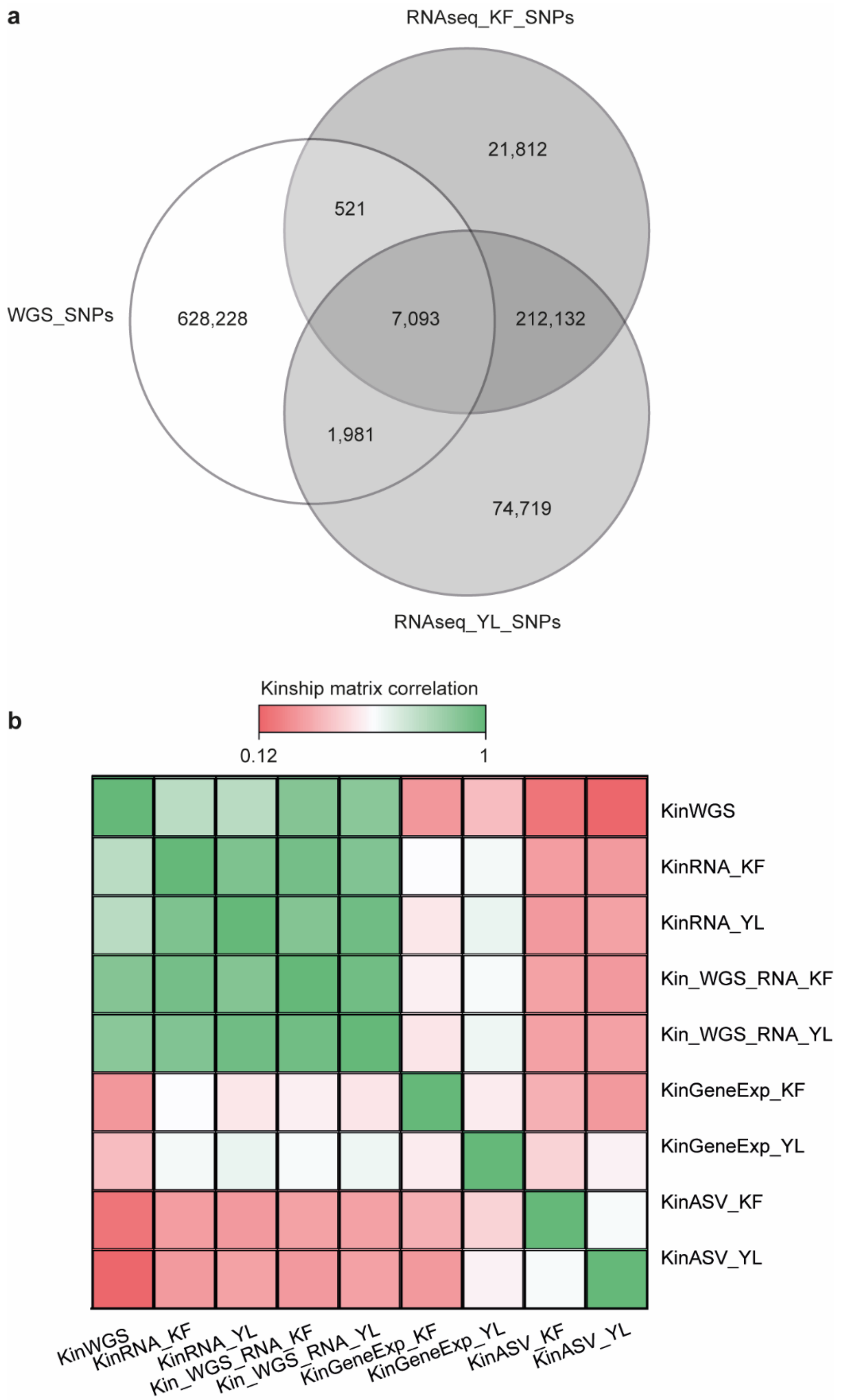

Supplemental Figure 5. Preparation of multi-omics datasets used for genomic prediction. (a) Venn diagram showing the overlap of SNPs derived from whole-genome sequencing (WGS) and RNA-seq–based SNP calling from the two field locations: Kaifeng (KF) and Yangling (YL). Numbers indicate the total count of SNPs unique to each dataset and those shared among datasets. SNP calling was performed once for each data type and location; no statistical tests were applied. (b) Heatmap displaying pairwise correlations among kinship matrices constructed using different omics datasets: WGS SNPs, RNA-seq SNPs from KF and YL, combined WGS+RNA-seq SNP sets, gene expression profiles, and ASV abundance profiles. The colour scale (0.12 to 1.0) represents the Pearson correlation between kinship matrices, where green denotes higher similarity and red denotes lower similarity. Each matrix was computed from the full set of available samples for that dataset (WGS: n = 175 ecotypes; RNA-seq:

n = 175 ecotypes per location; gene expression: n = 175; ASVs: n = 1341). No hypothesis testing was conducted beyond correlation estimation.

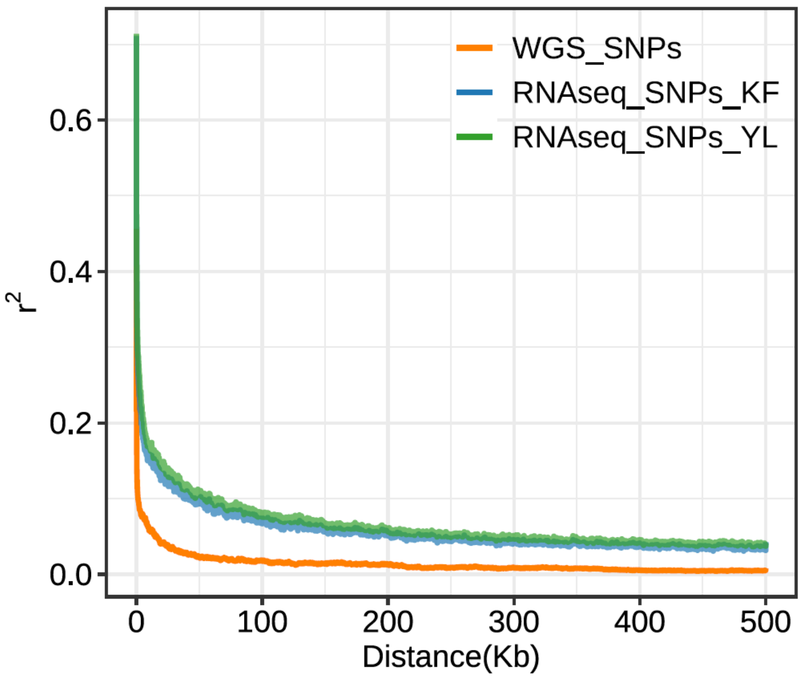

Supplemental Figure 6. Linkage disequilibrium (LD) decay across WGS- and RNA-seq–derived SNP datasets. LD decay plots showing the decline of pairwise linkage disequilibrium (r^2) with increasing physical distance (kb) between SNPs for whole-genome sequencing (WGS) SNPs and RNA-seq–derived SNPs obtained from Kaifeng (RNAseq_SNPs_KF) and Yangling (RNAseq_SNPs_YL) samples. Curves represent the mean r^2 values across all SNP pairs within each distance bin. LD was calculated genome-wide using bi-allelic SNPs filtered for minor allele frequency and missingness. No statistical hypothesis testing was applied for LD visualization. KF: Kaifeng; YL: Yangling.

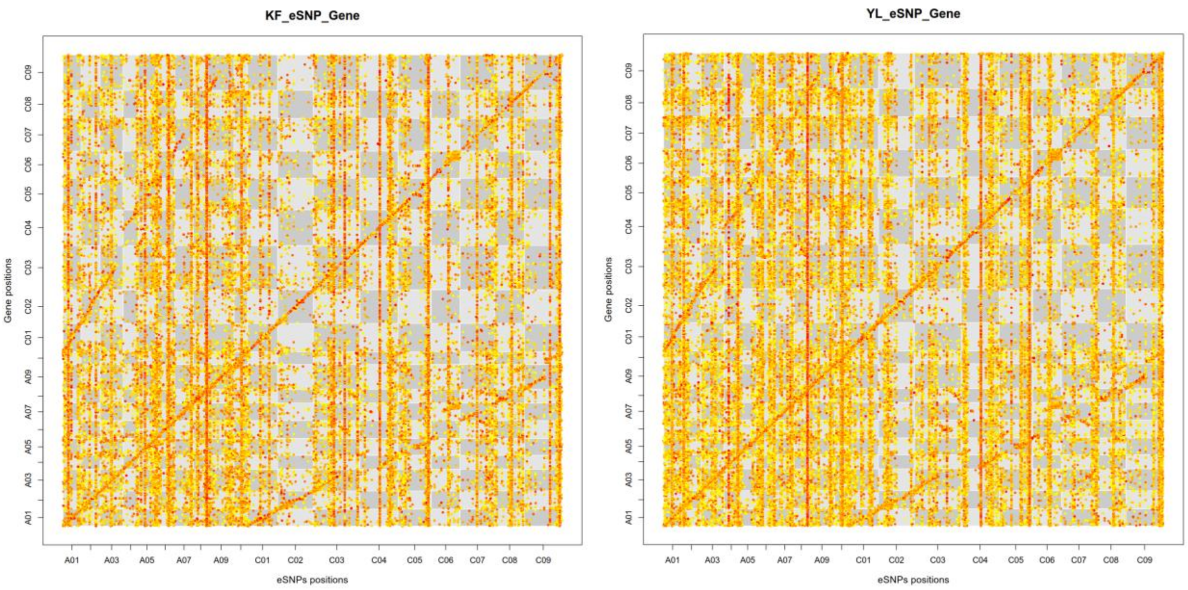

Supplemental Figure 7. Dot plot showing eQTLs and their regulated genes in 19 chromosomes. x-axis shows the single nucleotide polymorphism (SNP) position (bp) in each chromosome and the y-axis shows the gene position (bp) in each chromosome, with a chromosome order of A01 to C09 from left to right (x-axis) or from lower to upper (y-axis). The color of each dot represents the significance (P-value) of each eQTL-gene association, with low significance in yellow and high significance in red. Each chromosome is scaled by the physical chromosome length. Dots in the diagonal line show the intra-chromosomal associations. The lines show the enrichment of inter-subgenomic associations in homoeologous chromosomes and inter-chromosomal associations between the hotspot and other chromosomes. KF, Kaifeng; YL, Yangling.

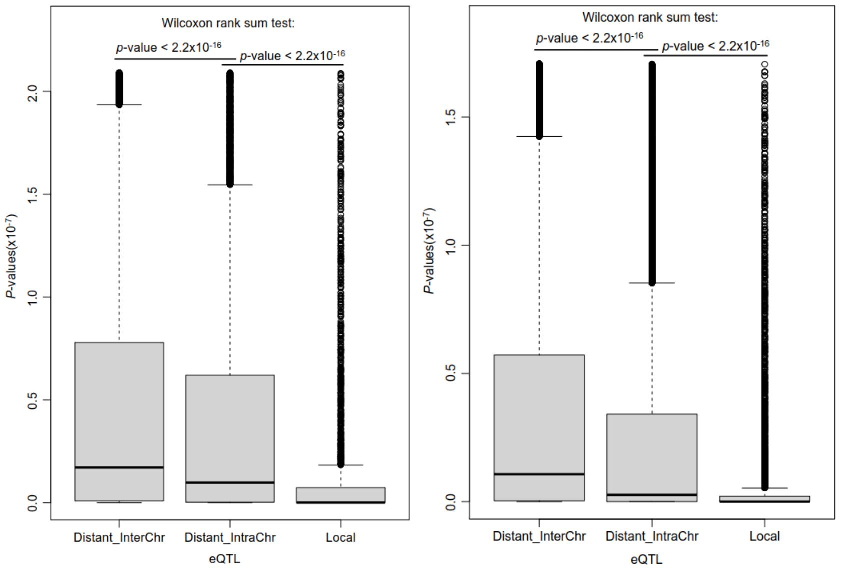

Supplemental Figure 8. The comparison of significance (*P* values) between inter-chromosomal eQTLs (Distant_InterChr) and intra-chromosomal eQTLs (Distant_IntraChr and Local) in KF (left) and YL (right). Boxplots show the distribution of $-\log_{10}(P)$ eQTL significance values for inter-chromosomal distant eQTLs (Distant_InterChr), intra-chromosomal distant eQTLs (Distant_IntraChr), and local eQTLs (Local) identified in Kaifeng (KF; left) and Yangling (YL; right). Each point represents one detected eQTL association (KF: n = 79,810 eQTLs; YL: n = 121,955 eQTLs). Statistical differences between categories were assessed using a two-sided Wilcoxon rank-sum test; the exact *P*-values are indicated above the comparisons ($P < 2.2 \times 10^{-16}$ for all pairwise tests). The y-axis denotes $-\log_{10}$ (P) of eQTL associations. No adjustments were applied for multiple testing in the visualization.

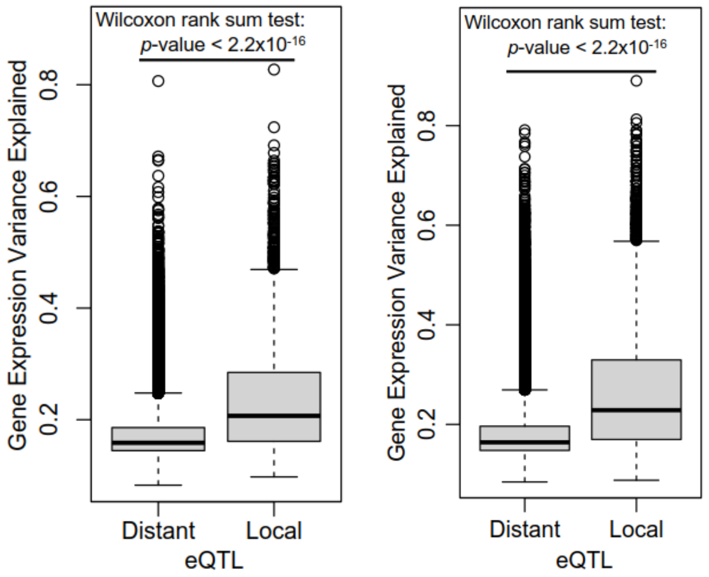

Supplemental Figure 9. Difference of gene explanation variance explained (R^2) of SNPs between local eQTL and distant eQTL in Kaifeng (left) and Yangling (right). Boxplots show the distribution of variance explained (R^2) by SNPs associated with local eQTLs and distant eQTLs for genes in Kaifeng (KF; left) and Yangling (YL; right). Each point represents one eQTL association (KF: n = 76,389 distant eQTLs, n = 3,421 local eQTLs; YL: n = 117,235 local eQTLs, 4,719 local eQTLs). Statistical differences between local and distant eQTLs were assessed using a two-sided Wilcoxon rank-sum test, with exact P-values indicated above the comparisons ($P < 2.2 \times 10^{-16}$ for both sites). The y-axis denotes the fraction of gene expression variance explained (R^2) by each SNP.

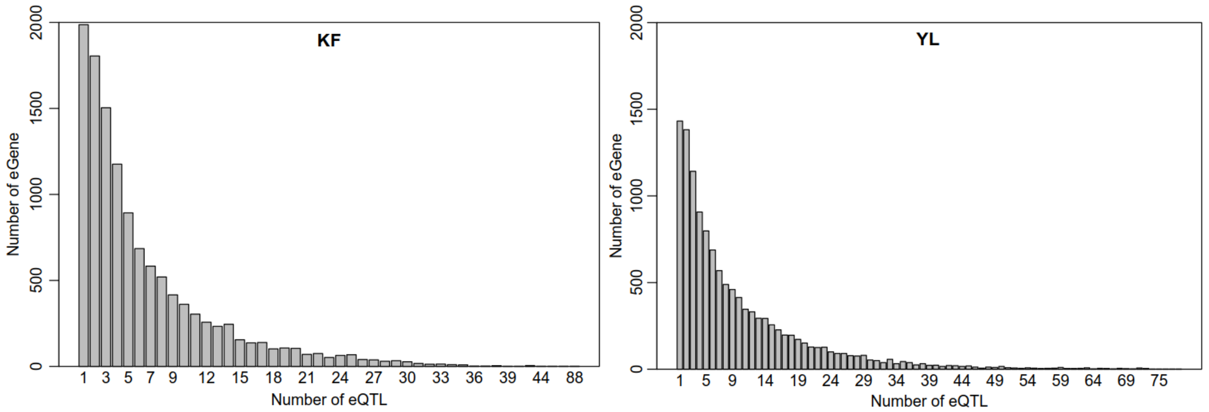

Supplemental Figure 10. Distribution of the number of eQTLs for genes which were regulated by eQTL in Kaifeng (left) and Yangling (right). Histograms show the frequency distribution of the number of eQTLs regulating each eGene identified in Kaifeng (KF; left) and Yangling (YL; right). Each bar represents the number of genes associated with a given number of detected eQTLs. Analyses were performed using all genes with at least one significant eQTL detected in each location (KF: n = 12,293 eGenes; YL: n = 12,322 eGenes). The x-axis indicates the number of eQTLs per gene, and the y-axis shows the number of eGenes in each category. No statistical hypothesis tests were applied for these descriptive distributions.

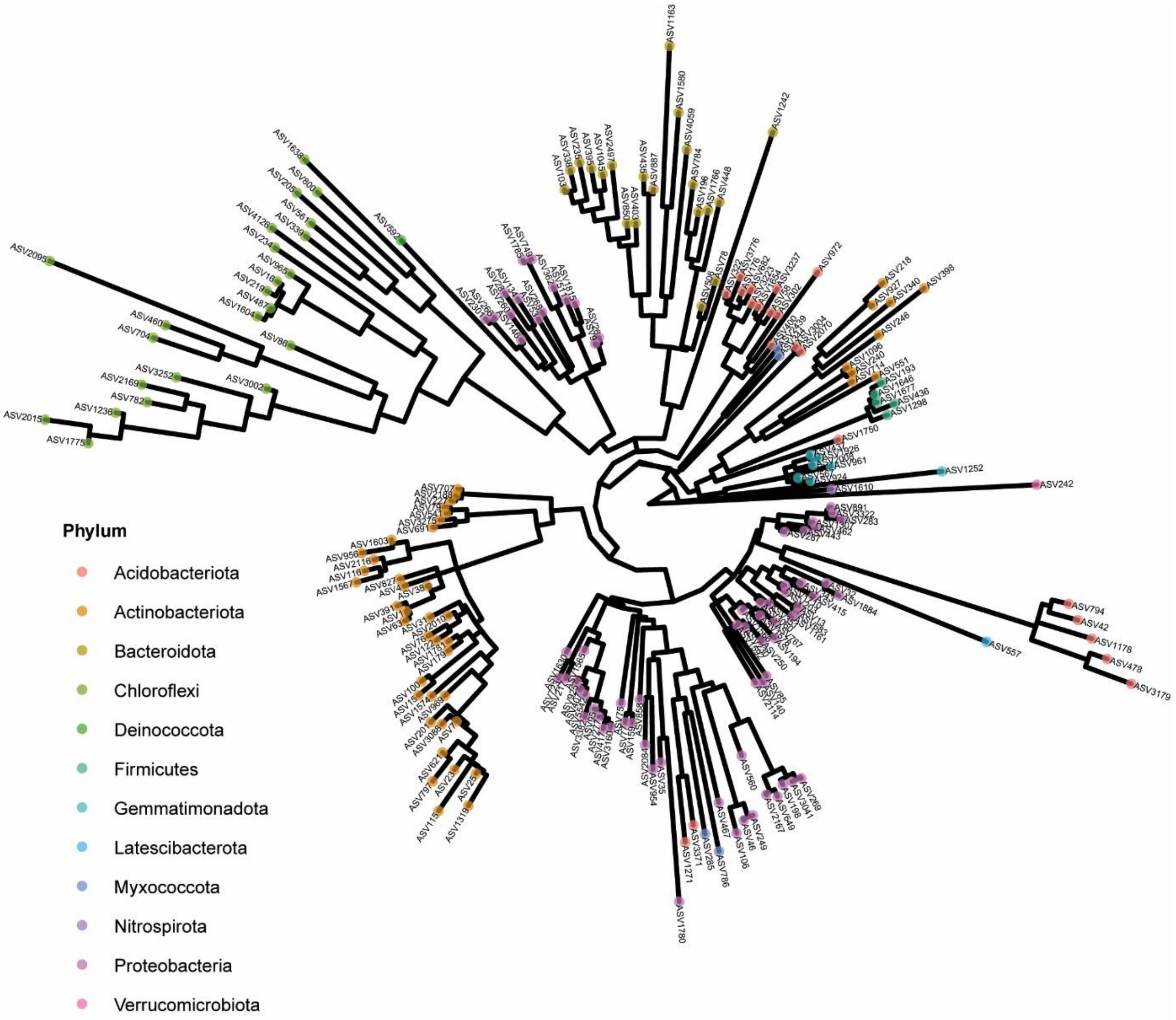

Supplemental Figure 11. Phylogenetic tree of 203 highly heritable ASVs. Circular phylogenetic tree showing the relationships among the 203 heritable bacterial ASVs (*n* = 203). ASVs are colour-coded according to their phylum-level taxonomic assignment, as indicated in the legend. The full list of ASVs and their taxonomic annotations is provided in Supplemental Dataset 3. No statistical tests were applied for this visualization; the tree was generated once from aligned ASV representative sequences.

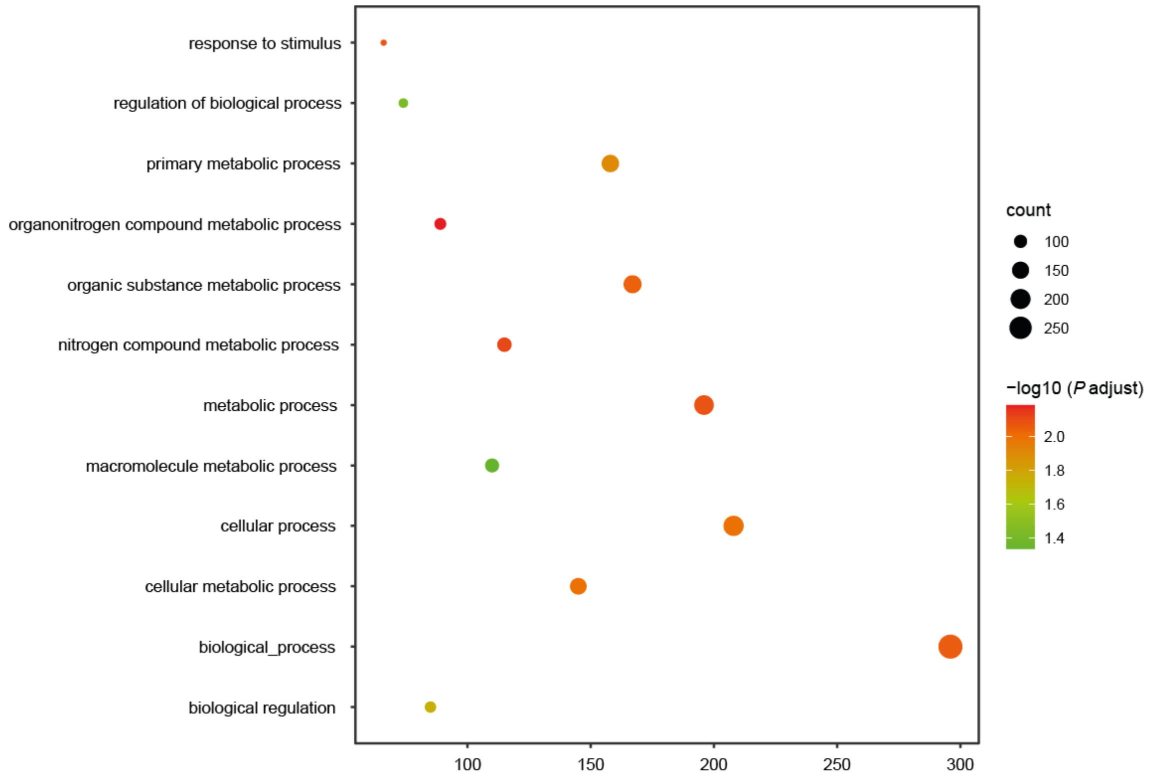

Supplemental Figure 12. Gene ontology (GO) analysis of ASV729 associated genes. Bubble plot showing the significantly enriched biological process GO terms among genes associated with ASV729. Each dot represents one GO term, where the dot size indicates the number of ASV-associated genes annotated to that term, and the colour scale (green to red) reflects the $-\log_{10}$(adjusted *P*-value) of enrichment. Enrichment significance was assessed using GO over-representation analysis with multiple-testing correction (Benjamini–Hochberg FDR). No additional statistical tests were applied for visualization.

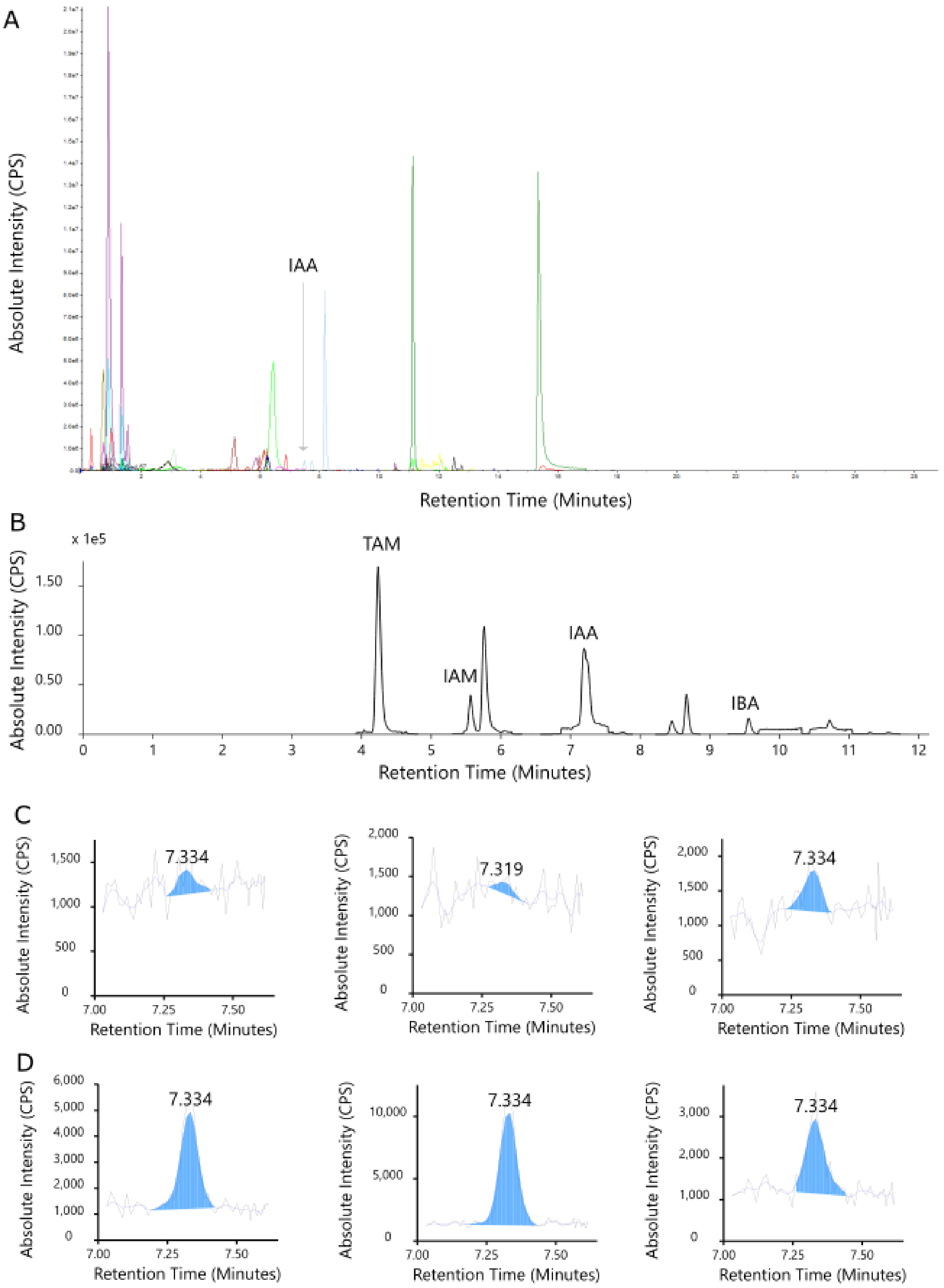

Supplemental Figure 13. Detection and quantification of indole-3-acetic acid (IAA) in rapeseed roots with and without *Sphingopyxis* inoculation using untargeted and targeted metabolomics approaches. (A) Representative extracted ion chromatograms (XIC) from untargeted LC-MS/MS analysis showing the putative detection of IAA (RT ≈ 7.30–7.35 min) in rapeseed root samples. The peak assignment is based on m/z, retention time, and MS/MS fragmentation matched to in-house libraries, consistent with standards used in the targeted assays. **(B)** Representative total ion

chromatogram (TIC) from targeted LC-MS/MS analysis showing separation and detection of key auxin-related metabolites, including tryptophan-derived intermediates: tryptamine (TAM), indole-3-acetamide (IAM), indole-3-acetic acid (IAA), and indole-3-butyric acid (IBA). Peaks were detected using MRM transitions in positive ion mode (e.g., IAA: 176→130). **(C)** Extracted ion chromatograms (XICs) for IAA (retention time ~7.33 min) in mock-treated root samples (M2-1, M2-2, M2-3), showing low signal intensity and consistent baseline. **(D)** XICs of IAA in roots inoculated with *Sphingopyxis* (M3-1, M3-2, M3-3), indicating elevated IAA accumulation upon bacterial treatment. Shaded regions represent the integrated peak area used for quantification. Retention time and fragmentation match were confirmed using authentic standards. See Supplementary Dataset 12 for detailed values and related information. No statistical hypothesis testing was applied to chromatographic peak display.

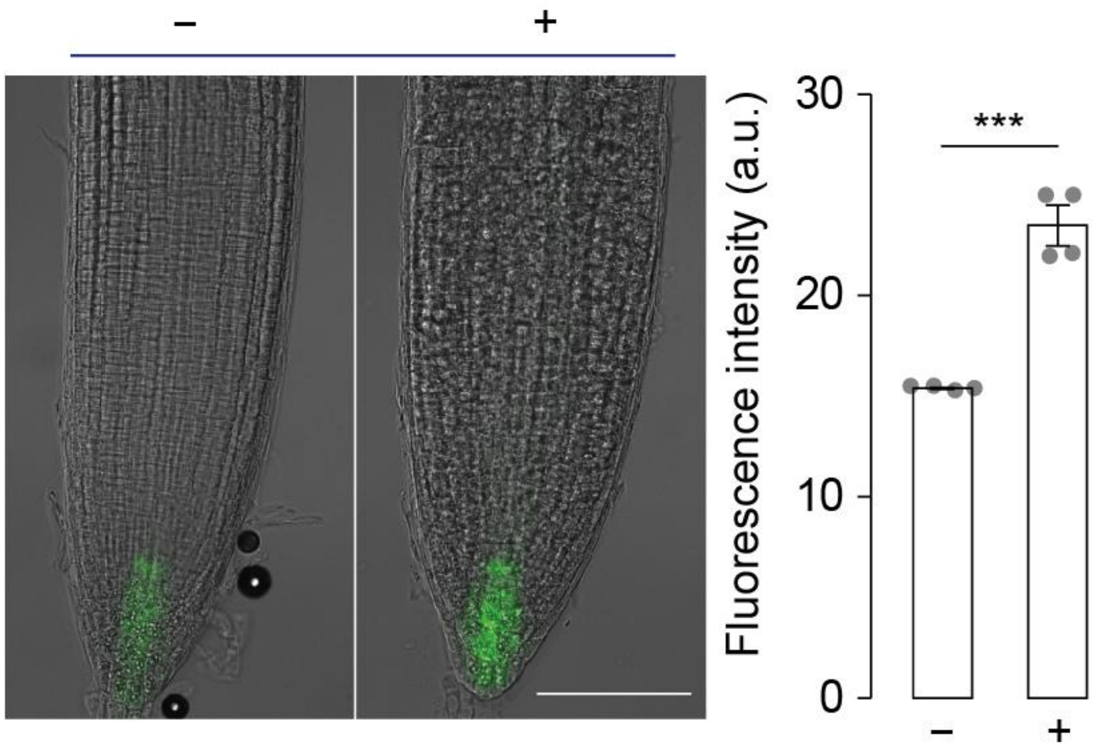

Supplemental Figure 14. DR5::GFP tagged root tip imaging with or without inoculation of *Sphingopyxis* isolate (29-6000-31). Representative confocal micrographs of root tips from DR5 reporter lines grown without (–) or with (+) inoculation of *Sphingopyxis* isolate 29-6000-31. Increased reporter signal intensity was consistently observed in inoculated roots. Scale bar = 100 µm. Quantification of fluorescence intensity (arbitrary units, a.u.) is shown on the right (n = 4 biological replicates per treatment). Data were analyzed using a two-sided paired Student's t-test; ****P* < 0.001. Each dot represents an individual root.

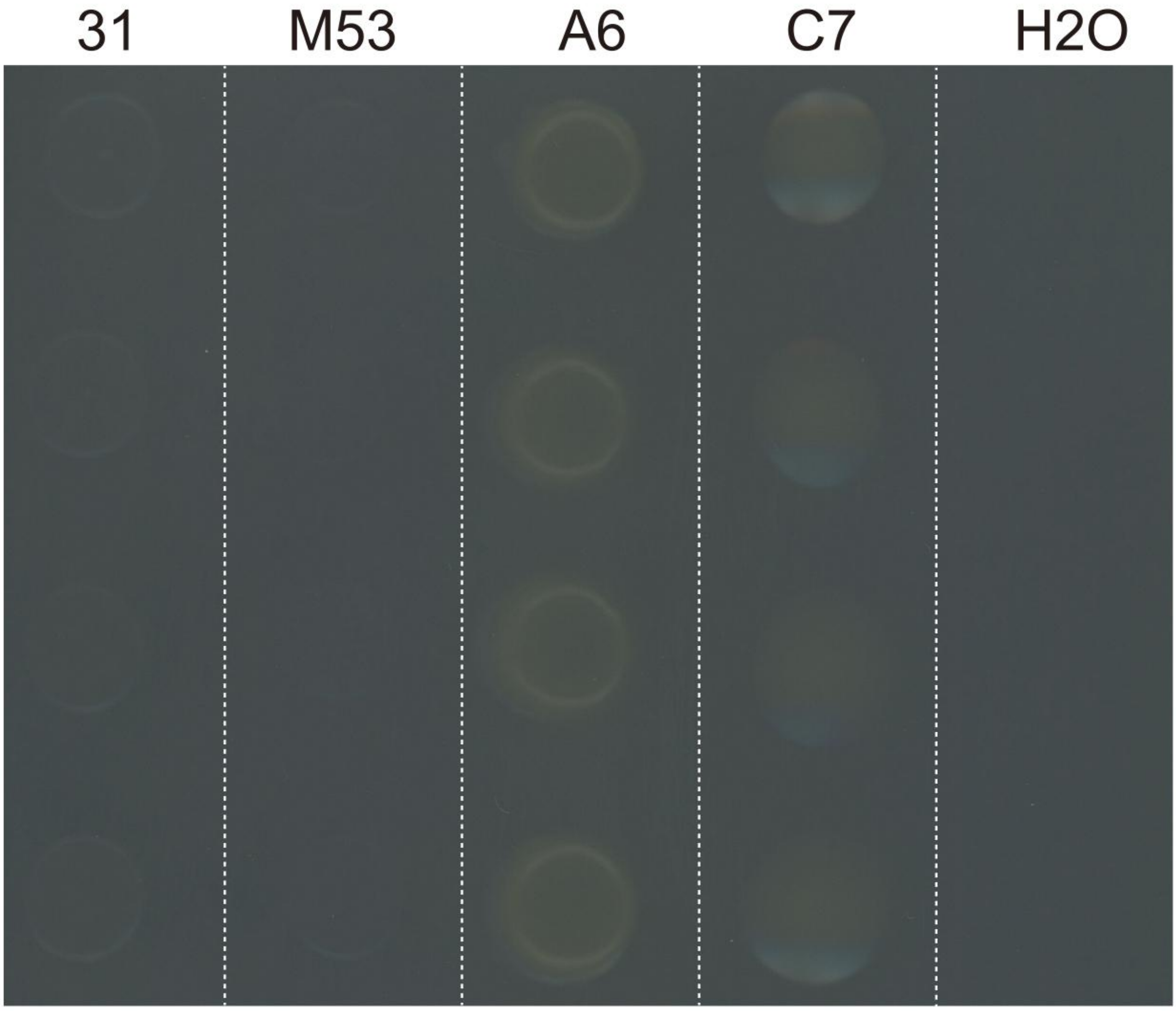

Supplemental Figure 15. Nitrogen-fixation capacity assay for *Sphingopyxis* isolates 31 and M53. Nitrogen-free growth assays showing the ability of bacterial isolates to grow on nitrogen-free medium. Isolates 31 and M53 showed no detectable colony formation, indicating a lack of nitrogen-fixing capacity. In contrast, two known nitrogen-fixing strains (A6 and C7) exhibited robust growth and served as positive controls, validating the assay. Sterile H_2O was used as a negative control.

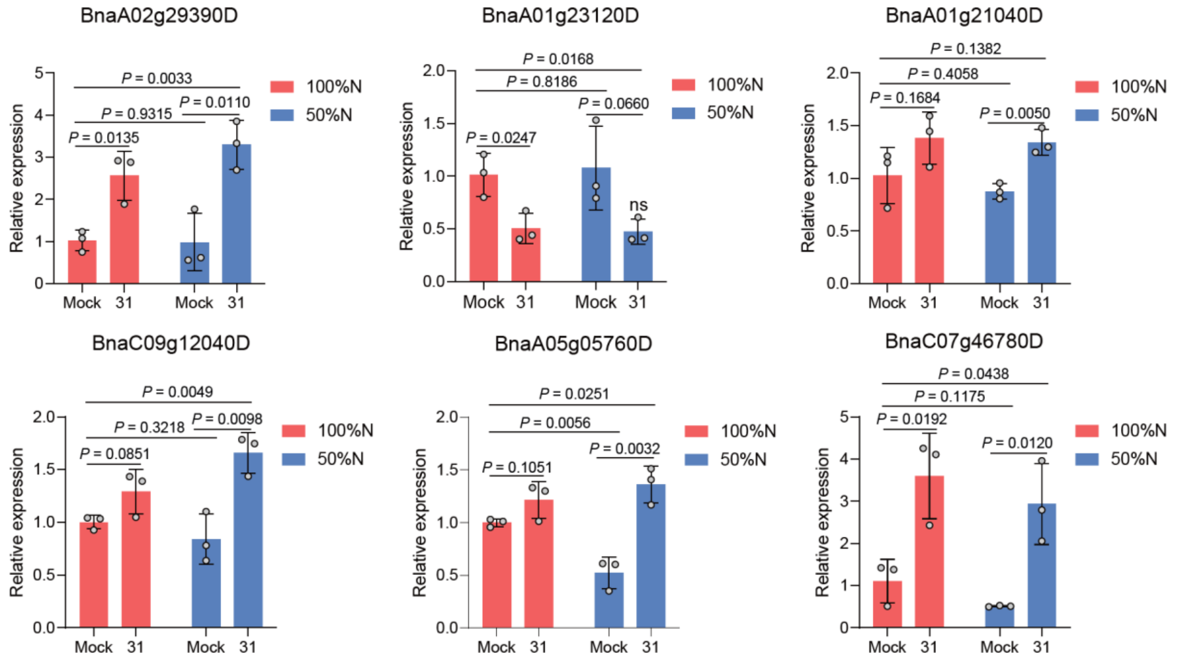

Supplemental Figure 16. qRT–PCR analysis of rapeseed gene expression following inoculation with *Sphingopyxis* isolate 31 under two nitrogen regimes. Relative expression levels of selected rapeseed genes were quantified in roots treated with mock or *Sphingopyxis* 31 under 100% N and 50% N nutrient conditions. Each bar represents the mean expression value, with dots indicating individual biological replicates (n = 3 per treatment). Statistical comparisons between treatments were performed using a two-sided paired Student's *t*-test, and exact *P*-values are shown above each comparison. Gene identifiers are displayed above each panel.

Supplemental Table 1 Local climate factors, physical and chemical properties of the basic soil at two locations. The values presented are mean ± standard error at Kaifeng (KF) and Yangling (YL) fields in 2019. Means followed by the same letter are not significantly different at $p < 0.05$ according to LSD (ANOVA, turkey HSD, $n = 5$).

**Soil properties**	**Kaifeng (KF)**	**Yangling (YL)**
Mean annual temperature (°C)	15.4	14
Annual precipitation (mm)	730	540
Total N (%)	0.094 ± 0.0027a	0.076 ± 0.00088b
Conc. P (mg/kg)	69.23 ± 1.61a	67.55 ± 1.06a
pH	6.74 ± 0.08b	7.23 ± 0.07a
Total organic carbon (%)	0.60 ± 0.062a	0.56 ± 0.043a
