# Supplementary material for: Large-scale multi-omics unveils host–microbiome interactions driving root development and nitrogen acquisition
Source: Nat Plants. 2026 Feb 3;12(2):319–36. doi: 10.1038/s41477-025-02210-7 (PMC12929062; doi:10.1038/s41477-025-02210-7)
Supplement: Supplementary file 2 — Reporting Summary [file 41477_2025_2210_MOESM2_ESM.pdf]

nature portfolio

Corresponding author(s): Daojie Wang, Yanfeng Zhang, Yang Bai and Peng Yu

Last updated by author(s): Nov 23, 2025

Reporting Summary

Nature Portfolio wishes to improve the reproducibility of the work that we publish. This form provides structure for consistency and transparency in reporting. For further information on Nature Portfolio policies, see our Editorial Policies and the Editorial Policy Checklist.

Statistics

For all statistical analyses, confirm that the following items are present in the figure legend, table legend, main text, or Methods section.

n/a	Confirmed	
☐	☒	The exact sample size (*n*) for each experimental group/condition, given as a discrete number and unit of measurement
☐	☒	A statement on whether measurements were taken from distinct samples or whether the same sample was measured repeatedly
☐	☒	The statistical test(s) used AND whether they are one- or two-sided<br>*Only common tests should be described solely by name; describe more complex techniques in the Methods section.*
☒	☐	A description of all covariates tested
☐	☒	A description of any assumptions or corrections, such as tests of normality and adjustment for multiple comparisons
☐	☒	A full description of the statistical parameters including central tendency (e.g. means) or other basic estimates (e.g. regression coefficient) AND variation (e.g. standard deviation) or associated estimates of uncertainty (e.g. confidence intervals)
☐	☒	For null hypothesis testing, the test statistic (e.g. *F*, *t*, *r*) with confidence intervals, effect sizes, degrees of freedom and *P* value noted<br>*Give P values as exact values whenever suitable.*
☐	☒	For Bayesian analysis, information on the choice of priors and Markov chain Monte Carlo settings
☒	☐	For hierarchical and complex designs, identification of the appropriate level for tests and full reporting of outcomes
☐	☒	Estimates of effect sizes (e.g. Cohen's *d*, Pearson's *r*), indicating how they were calculated

Our web collection on statistics for biologists contains articles on many of the points above.

Software and code

Policy information about availability of computer code

Data collection	N/A
Data analysis	For microbiome data, paired-end reads were merged using FLASH (v1.2.11), quality filtered with fastp (v0.19.6), sequence analyses were performed by QIIME 2 software (v2020.2) and denoised with q2-dada2 (via QIIME 2). ASVs was performed using the sklearn-based Naive Bayes classifer implemented in Qiime2 and the SILVA 16S rRNA database (v138). Shannon index was calculated with Mothur (v1.30.1). Principal coordinate analysis (PCoA) based on Bray-Curtis dissimilarity using R Vegan (v2.4-3) package.<br>Raw RNAseq paired end reads were trimmed and quality controlled by fastp (V0.19.5) and sequences mapped using HISAT2 (V2.1.0). RSEM (V 1.3.1) was used to quantify gene expression. WGCNA (V1.72.1) was used to identify different gene expression modules. Network visualization was performed in Cytoscape (V3.8.0) only for significant modules. All linear mixed models were fitted using the software ASReml-R 4.0. Genotypic data was imputed by Beagle v5.2. eQTL hotspots were visualized using R package circlize v0.4.13. QTL, Hotspot, Gene and ASV were visualized using Gephi v0.10. The Pearson correlation method was used in 'mantel' function in vegan package (v2.6.4) of R. GO enriched terms were identified using Goatools. Bioinformatic analyses for bacterial whole genome were performed using the Majorbio Cloud (http://cloud.majorbio.com). Whole genome short and long reads were co-assembled to construct complete genomes using Unicycle (v0.4.8). Unicycler uses Pilon (v1.22) was used to polish the assembly. The coding sequences (CDs) of chromosome and plasmid were predicted using Prodigal (v2.6.3). tRNA-scan-SE (v 2.0) was used for tRNA prediction and Barrnap (v0.9). The predicted CDs were annotated from COG (https://www.ncbi.nlm.nih.gov/research/COG) and KEGG database (https://www.genome.jp/kegg/kegg1.html) using sequence alignment tools Diamond and HMMER. HPLC-MS/MS generated data files were processed with the SCIEX OS Version 1.4.

For manuscripts utilizing custom algorithms or software that are central to the research but not yet described in published literature, software must be made available to editors and reviewers. We strongly encourage code deposition in a community repository (e.g. GitHub). See the Nature Portfolio guidelines for submitting code & software for further information.

Data

Policy information about availability of data

All manuscripts must include a data availability statement. This statement should provide the following information, where applicable:

- Accession codes, unique identifiers, or web links for publicly available datasets
- A description of any restrictions on data availability
- For clinical datasets or third party data, please ensure that the statement adheres to our policy

All raw rapeseed RNA-seq and bacterial 16S gene data generated in this study have been deposited in the Sequence Read Archive (http://www.ncbi.nlm.nih.gov/sra) under the BioProject IDs: PRJNA986524 (KF, RNA-seq), PRJNA990484 (YL, RNA-seq), PRJNA956663 (KF, 16S) and PRJNA960662 (YL, 16S). Raw format mzML and processed metabolomics data—including peak tables, compound annotations (MSI Level 2), and quantification results from both untargeted and targeted assays — are deposited under the link https://figshare.com/s/caa71efed449b6f8c4e0. All data necessary to reproduce the results are included in the processed datasets, and additional materials can be made available upon reasonable request. We deposited customized scripts in the following GitHub repository: https://github.com/Ligl0226/proj_rapeseed.

Human research participants

Policy information about studies involving human research participants and Sex and Gender in Research.

Reporting on sex and gender	N/A
Population characteristics	N/A
Recruitment	N/A
Ethics oversight	N/A

Note that full information on the approval of the study protocol must also be provided in the manuscript.

Field-specific reporting

Please select the one below that is the best fit for your research. If you are not sure, read the appropriate sections before making your selection.

☒ Life sciences ☐ Behavioural & social sciences ☐ Ecological, evolutionary & environmental sciences

For a reference copy of the document with all sections, see nature.com/documents/nr-reporting-summary-flat.pdf

Life sciences study design

All studies must disclose on these points even when the disclosure is negative.

Sample size	No specific statistical methods were used to determine sample size for the field experiments. Instead, 300 ecotypes of B. napus were grown in the field. All of these genotypes fully reflect B. napus genetic diversity, were chosen for our main study. All detailed information was provided with supplemental dataset and figures.
Data exclusions	We have performed the outlier detection analysis using specific model and have not found any outliers in our dataset. (See Methods)
Replication	The main pot experiment for microbiome sequencing was performed once with three independent biological replicates and each replicate included three randomly selected plant roots. The soil inoculation experiment was performed for three dependent replicates in both soil and agarose system. All of them were successfully performed.
Randomization	Different B. napus ecotypes were grown in each field with a randomly complete block design.
Blinding	All rhizosphere samples were harvested blindly with only samples numbers without access to the genotype identities. Data analysis was blinded to genotypes and allowing them to choose each subject blindly. Data on biomass, nutrients analysis were acquired by personnel without genotype information.

Reporting for specific materials, systems and methods

We require information from authors about some types of materials, experimental systems and methods used in many studies. Here, indicate whether each material, system or method listed is relevant to your study. If you are not sure if a list item applies to your research, read the appropriate section before selecting a response.

Materials & experimental systems

n/a	Involved in the study
☒	☐ Antibodies
☒	☐ Eukaryotic cell lines
☒	☐ Palaeontology and archaeology
☒	☐ Animals and other organisms
☒	☐ Clinical data
☒	☐ Dual use research of concern

Methods

n/a	Involved in the study
☒	☐ ChIP-seq
☒	☐ Flow cytometry
☒	☐ MRI-based neuroimaging
